# Humanistic burden of pediatric type 1 diabetes on children and informal caregivers: systematic literature reviews

**DOI:** 10.1186/s13098-024-01310-2

**Published:** 2024-03-21

**Authors:** Veleka Allen, Aymeric Mahieu, Ellen Kasireddy, Walid Shouman, Mir-Masoud Pourrahmat, Jean-Paul Collet, Andriy Cherkas

**Affiliations:** 1 Sanofi, Bridgewater, NJ USA; 2 Sanofi, Paris, France; 3 Evidinno Outcomes Research Inc., Vancouver, BC Canada; 4 Sanofi, Frankfurt am Main, Germany

**Keywords:** Type 1 diabetes, Informal caregiver, Humanistic burden, Systematic literature review, Conceptual framework

## Abstract

**Background:**

Diagnosis of children with type 1 diabetes (T1D) imposes an unprecedented burden on children and their caregivers.

**Objective:**

To assess the burden of T1D on children and their informal caregivers, both after a recent diagnosis or after a longer duration of disease.

**Methods:**

A series of systematic literature reviews were performed to explore the burden of T1D on children with the disease and their primary informal caregivers, based on the time of diagnosis. After the extraction of the qualitative and quantitative data from the included studies, two literature-based conceptual frameworks were developed: on the burden of pediatric T1D on children, and on informal caregivers. A third conceptual framework on the shared burden of pediatric T1D on both children and informal caregivers as part of the same family unit was also developed.

**Results:**

The review of literature has identified a series of factors that affect the quality of life of children with T1D and their informal caregivers, with a direct impact on physical, emotional, and social outcomes. Generally, female patients and older adolescents experience more worry and stress that affects their quality of life. Other categories of factors affecting the child’s and caregiver’s burden include social, emotional, and physical factors, treatment-related and disease-related factors, as well as their coping abilities. Anxiety, depression, stress, and worry were commonly found among children and caregivers, starting with the diagnosis of T1D and continuing over time in relation to new challenges pertaining to aging or the disease duration.

**Conclusion:**

T1D causes a significant burden to affected children and their caregivers, both independently and through transactional interaction within the family unit. Disease burden can be reduced by strengthening individuals for the benefit of the whole family.

**Supplementary Information:**

The online version contains supplementary material available at 10.1186/s13098-024-01310-2.

## Background

T1D is a chronic disease that is incurable and needs nearly permanent attention due to frequent injections or dose adjustments, the use of devices to monitor blood glucose, and different applications related to T1D management [1]. Having a child diagnosed with type 1 diabetes (T1D) can have a major impact and lifelong consequences on the affected individual and their family. Moreover, T1D often leads to long-term complications such as heart disease, chronic kidney disease, nerve damage, vision and hearing problems, as well as mental health problems [2]. T1D is associated with hyperglycemia and a risk of hypoglycemia, a relatively common complication in children treated with an intensive insulin regimen. Families of children with T1D strive to manage both hypo and hyperglycemia, as both can negatively affect the child’s cognitive skills [3]. In children, the episodes of hypoglycemia associated with seizures require careful monitoring to ensure that they are not adversely affecting learning [4]. Finally, there is an economic impact on the family caused by the child’s T1D diagnosis. This is not only due to the direct costs of treatment but also to the dietary and lifestyle changes and potential negative impact on the caregiver’s professional life, that the family must undergo.

People with T1D have been reported to score lower across all health-related quality of life (QoL) measures compared to their unaffected peers [5]. Additionally, people with T1D, particularly children, require significant caregiving. As T1D is a chronic disease with currently no cure, immediate family members (e.g., parents in the case of children with T1D) often provide informal care, which is often associated with high levels of psychological distress [6]. This is particularly significant in the early period after diagnosis, as disease onset is often sudden and unexpected [7]. Furthermore, the negative impact on psychological health can sometimes be larger for family members than for patients [8]. Psychological health challenges can therefore be considered contagious within the family [8]. Parent-child interactions in the context of T1D tend to be poor [9], and sharing the diabetes responsibility and diabetes-related family conflict contribute to the burden on the whole family [10].

In this context, this review aimed to summarize the available evidence on the humanistic burden of T1D on children, adolescents, and young adults (aged 6 to 21 years old) and their informal caregivers as part of a family unit [7]. Therefore, we conducted a series of systematic literature reviews (SLRs) to assess the burden of T1D diagnosis on children and their informal caregivers, both after a recent diagnosis or after a longer duration of disease. Additionally, we focused on the elements of distress that children with T1D and informal caregivers have in common to identify the shared burden in the context of the family unit. To our knowledge, this specific review, which focused on the shared burden of children and parents or other informal care providers when facing T1D adversity, is the first of this type in the published literature.

## Materials and methods

### Conducting the systematic literature reviews

We performed a series of SLRs to explore the burden of T1D on children with the disease and their primary caregivers, based on the time of diagnosis (recently diagnosed or a longer duration of disease). Informal caregivers were defined as people who take care of children with T1D without payment, which can be immediate and extended family members, or other persons providing care voluntarily. The current review is focused on informal caregivers and does not include professional caregivers or healthcare providers. In our review, a recent diagnosis was defined as a diagnosis of T1D made within three months.

The World Health Organization considers children as individuals who are younger than 18 years of age; adolescents as individuals in the 10–19 years age group; and youth as individuals in the 15–24 years age group [11]. The population of interest included children, adolescents, and young adults between 6 and 21 years old and diagnosed with T1D. This age range was set to limit the heterogeneity of the included patients; children between 6 and 21 are generally in a relatively similar setting regarding having a close relationship with parents and attending school. Children younger than 6 years of age can be in a variety of settings (e.g., home, daycare, preschool), and they also have more difficulty expressing their quality of life and answering questions independently. Similarly, young adults older than 21 can be at university, in the workforce, or both, and most likely have become independent in managing their T1D. This 6–21 years age range also allows us to investigate patients during a difficult period of glycemic control because, during puberty, patients have increased pubertal hormone levels, which requires more follow-up and higher doses of treatment [12]. During puberty, adolescents also have behavioral changes, where they become rebellious and tend to not follow rules [13].

Study identification and eligibility criteria (see Additional File 1) were developed using the Population, Intervention, Comparator, and Outcome (PICO) framework as described by the Cochrane Handbook for Systematic Reviews of Interventions [14]. Relevant publications were identified by searching MEDLINE® and Embase via OvidSP, and PsycINFO via EBSCOhost using predefined search strategies (see Additional File 2). Abstracts from relevant conferences were searched within the last two years via Embase. Results for the review were reported according to the Preferred Reporting Items for Systematic Reviews and Meta-Analyses (PRISMA) guidelines [15].

### Development of the literature-based conceptual frameworks

In addition to the series of SLRs, two literature-based conceptual frameworks were developed to illustrate the humanistic burden of T1D on children and their caregivers: (i) a conceptual framework on the burden of pediatric T1D on children, and (ii) a conceptual framework on the burden of pediatric T1D on informal caregivers. For the development of the conceptual frameworks, quantitative and qualitative data from the included studies were extracted.

A list of concepts from the extracted data was created, and the number of studies that reported on each of the concepts was counted. To make the conceptual frameworks more explicit, the responses to T1D occurrence were grouped under three main overarching themes related to quality of life: emotional/mental response-theme, physical response-theme, and social response-theme, with various elements in each of them. The decision to individualize these three themes is grounded in the recognition of their importance and interconnectedness to affect health and wellbeing [16] and the implications they have for individuals and caregivers. Emotional responses, such as stress and anxiety, involve complex psychological processes and require specific interventions to address mental well-being. Meanwhile, social responses encompass interactions with others, social support, and societal attitudes, which influence social functioning and quality of life. Finally, physical responses involve physiological effects, symptoms, and medical management, necessitating interventions tailored to symptom management and lifestyle modifications. These three domains are strongly related to each other as excess stress may decrease social abilities, or a decrease in physical health may be a source of stress and social isolation. Most quality-of-life scales assess the impact on physical, social, and emotional aspects. For instance, SF-36 covers these themes with five subscales and also includes pain, general health, and vitality that were not selected in our project. By maintaining separate categories for the three domains, healthcare professionals can conduct a more comprehensive assessment of T1D burden and implement tailored interventions that address the diverse needs of individuals and caregivers in each domain, ultimately improving outcomes and enhancing overall well-being and self-confidence.

The next step was to identify the factors related to the three main burden-response themes to T1D occurrence. These factors were grouped into various categories: (i) sociodemographic, (ii) physical, (iii) disease-related, (iv) treatment-related, (v) social consequences, (vi) coping techniques, and (vii) emotional consequences. We also counted the number of times each factor was mentioned in the literature in relation to the three burden-response themes.

Another conceptual framework was developed to better understand the shared burden that pediatric T1D occurrence has on both children and informal caregivers, as part of the family unit. This shared conceptual framework focused on the factors that were found among both children with T1D and their caregivers.

It is worth mentioning that the developed frameworks only included factors that affected the QoL of patients and caregivers from the conducted SLRs. Moreover, these associations do not suggest causality and the straight lines within the graphic representations only show that these factors had been found associated with the burden due to T1D diagnosis.

## Results

In this section, we first present the burden of T1D on children who have been newly diagnosed within three months and children with any duration of disease. Similarly, we also describe the burden of T1D on parents/informal caregivers of these children. The third part describes the types of burden that are similar for children and their caregivers.

### Disease Burden in Children and Young adults with T1D

Two SLRs were conducted to describe the level of burden in children and young adults who were recently diagnosed and in those with T1D of any duration, respectively. Five studies reported the burden of pediatric T1D on children who were recently diagnosed with T1D, while 76 studies reported the burden in children with any duration of T1D (see the list of included studies in Additional File 3). We identified a wide variety of measures that were used to assess the burden of disease, including both quantitative and qualitative measures. A summary of the findings is presented in Table 1.

**Table 1:** Summary of outcomes in studies that assess the burden of T1D on children

Tool	Measure	Main Findings
Pediatric Quality of Life Inventory (PedsQL 4.0) [17, 39–48]	Overall QOL (Physical health, psychological health)	• Adolescents showed worse QoL than younger children. • Gender was a significant predictor of overall QoL, with boys experiencing better QoL. • School functioning was better for boys. • Adolescent girls experienced worse QoL as they grew older.
Pediatric Quality of Life Inventory (PedsQL 3.0) [18–21, 24, 40, 44, 47, 49–65]	Diabetes QoL (Diabetes symptoms, treatment barriers, treatment adherence, worry, communication)	• Adolescents experienced worse QoL. • Gender was a predictor, with girls having worse QoL than boys. • Patients with more depressive symptoms showed worse scores and worse QoL. • Acceptance of diagnosis and adherence to therapy led to better QoL. • Patients using CSII experienced better QoL than patients using MDI. • Patients using primary control coping methods showed better QoL.
Diabetes Quality of Life for Youth (DQOLY) [22, 23, 36, 66–70]	Overall QoL (Impact of diabetes, worry about diabetes, health perception, life satisfaction)	• Boys experienced better QoL than girls, in general. • Girls experienced more worry and reported lower self-rated health. • Adolescents experienced worse QoL when compared to younger patients. • Patients who desire to decrease their weight experienced worse QoL. • Patients who accept their diagnosis and integrate diabetes within their self-identity have better QoL and greater life satisfaction. *Recently diagnosed patients* [71] • Children (10 years and older) diabetes-related worries decreased over the first 9 months of diagnosis. • Impact of diabetes and satisfaction of life score showed an improvement in patients over the 9 months of diagnosis.
Children’s Depression Inventory (CDI) [24–26, 48, 54, 55, 72, 73]	Depression	• More female patients experienced depression than male patients. • Adolescents experienced more depression than younger children. *Recently diagnosed patients* [74] • Children (age 8–13) showed elevated levels of depression at diagnosis which significantly decreased 12 months after diagnosis.
Self-Care Inventory-Revised (SCI-R) [36, 57, 62, 69]	Perceived adherence to self-care regimen	• Children who accept their diabetes diagnosis and integrate within their self-identity were more confident and showed better self-care. • Patients who use an insulin pump showed better self-care than patients who use injectables. • Patients who had a high acceptance of the diagnosis and high adherence to treatment showed better self-care.
State-Trait Anxiety Inventory for Children (STAIC) [54, 62, 75, 76]	State anxiety (at the moment) and trait anxiety (in general)	• Patients had moderate state and trait anxiety.
Problem Areas in Diabetes for Pediatrics (PAID-Peds) [53, 64, 77]	Burden related to typical problems and issues in diabetes management	• Patients with moderate to severe depression showed a higher burden. • Patients with high resilience showed lower burden levels. • Issues that were experienced most by patients included friends or family not understanding the difficulty of diabetes, friends or family acting like a “diabetes police”, worrying about the future and complications, and experiencing interference in having fun with friends.

Results were obtained from studies reporting the burden of T1D on children recently diagnosed (< 3 months) and studies at any duration of disease. CSII: Continuous Subcutaneous Insulin Infusion; MDI: Multiple Daily Injections; QOL: Quality of Life

**Fig. 1:**
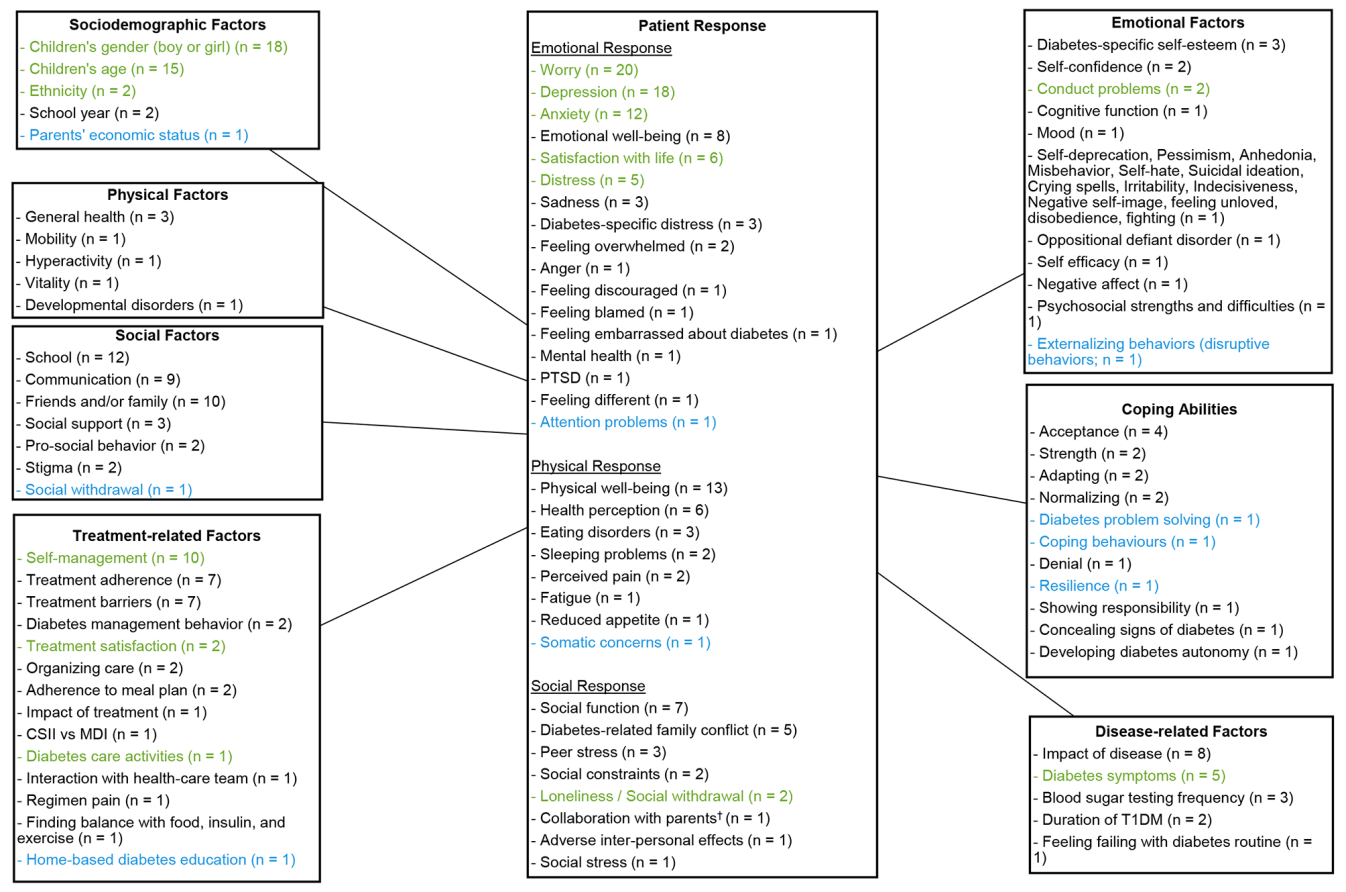
Literature-based conceptual framework on burden of T1D on children Elements in blue-colored font were found only in children who were recently diagnosed with T1D; elements in black-colored font were found only in children with T1D for at least three months; elements in green-colored font were found in children with T1D of any duration (both recently diagnosed and after three months of diagnosis

The review of the literature has identified a series of factors that affect the QoL of children, adolescents, and young adults with T1D, with a direct impact on physical, mental, and social outcomes. Elements associated with the child’s burden were grouped into seven different categories that included each several specific factors (Fig. 1): (i) Sociodemographic category includes the following factors: age, gender, and school year; (ii) Physical category includes factors such as general health and mobility; (iii) Disease-related category includes the impact of disease, blood sugar testing frequency, and duration of T1D; (iv) Treatment-related category includes factors like treatment adherence, treatment barriers, and diabetes management behavior; (v) Social category includes six factors: function at school, communication, friends and family, psychosocial aspect, social support, pro-social behavior, and stigma; (vi) the category of coping abilities includes a few essential factors: acceptance, strength, adapting, and normalizing; finally, (vii) the overarching category of emotional consequences includes diabetes-specific self-esteem and self-confidence-related factors.

These seven categories of factors have an impact on the child in the context of T1D. The overall impact on the child (or patient response) was further categorized into three types of expression: emotional response, physical response, and social response.

Some of the above factors are specific to newly diagnosed children (Fig. 1 - blue color), including the parent’s economic status, social withdrawal, and disruptive behavior, while other factors are specific to children who have been diagnosed with T1D for at least three months (Fig. 1– black color), such as diabetes-specific self-esteem, acceptance, impact of disease, and treatment adherence. Additionally, other factors were reported by children with any duration of T1D (i.e., both recently diagnosed and after three months of diagnosis), indicating that these factors could be experienced throughout the patient’s journey with T1D (Fig. 1– green color).

### Disease Burden in Informal caregivers of children with T1D

Two SLRs were conducted to describe the level of burden in caregivers of children who were recently diagnosed and in caregivers of children with T1D of any duration, respectively. Five studies reported the burden on caregivers of children with recently diagnosed T1D, while 51 studies reported the level of burden on caregivers of children with any duration of disease (see the list of included studies in Additional File 3). Table 2 summarizes the main outcomes found in the included studies.

**Table 2:** Summary of outcomes from included studies reporting the burden of pediatric T1D on informal caregivers

Tool	Measure	Main Findings
Hypoglycemia Fear Survey for Parents (HFS-P) [78–81]	Fear of hypoglycemia in parents of children with T1D	• Mothers showed higher HFS-P total scores and higher Worry subscale scores than fathers. • Parents of children younger than 12 years showed higher levels of fear of hypoglycemia when compared to parents of children 12 years or older. • Parents who reviewed their child’s blood glucose regularly had higher levels of fear of hypoglycemia. • Parents of children receiving CSII reported significantly reduced fear of hypoglycemia at 6 months follow-up when compared to the MDI group. *Recently diagnosed children* [82] • At diagnosis, caregivers of children aged 11 or younger had elevated levels of fear, and it decreased over time. • Parents using Parent Education Through Simulation–Diabetes had higher levels of fear.
Center for Epidemiological Studies– Depression Scale (CES-D) [55, 83–86]	Depression	• Parents did not experience clinical depression. • Parents, family members, or other informal caregivers of patients attending team clinical visits and those attending regular face-to-face visits did not show any significant changes in the CES-D scores over time and they all did not experience clinical depressive disorder.
Problem Areas in Diabetes Survey– Parents (PAID) [83, 87–91]	Diabetes-specific emotional distress in parents of youth with T1D	• Parents who use CGM showed lower emotional distress than parents who do not use technology.
Diabetes Family Conflict Scale (DFCS) [55, 92–94]	Family conflict around diabetes management	• Parents of patients with elevated risk of future complications showed the highest level of family conflict. • Family conflict is representative of the quality of parent-child relations, which is also predictive of glycemic control. • There was a decrease in family conflict in families using the Diabetes Learning Family Intervention (DEFLIN).
State-Trait Anxiety Inventory (STAI) [80, 83, 85, 86, 95, 96]	State anxiety (at the moment) and trait anxiety (in general)	• Mothers showed higher levels of state and trait anxiety, as well as overall anxiety. • Parents experienced more anxiety than their children.
Parenting Stress Index (PSI) [97]	Distress associated with the parental role	• Parents of adolescents (13–18 years) had higher levels of distress when compared with parents of younger children (8–12 years).
Zarit Burden Interview (ZBI) [98, 99]	The subjective psychological burden associated with providing care to a child with T1D	• Most parents reported experiencing moderate subjective burden.
Depression Anxiety and Stress Scale (DASS) [100–102]	Depression, anxiety, and stress	• Parents receiving stress management education experienced a decrease in the levels of stress, anxiety, and depression over time. • There was a decrease in stress, anxiety, and depression in parents using the Diabetes Learning Family Intervention (DEFLIN).

Results were obtained from studies reporting the burden of T1D on caregivers of children recently diagnosed (less than 3 months) and studies at any duration of disease. CSII: Continuous Subcutaneous Insulin Infusion; MDI: Multiple Daily Injections; QOL: Quality of Life

**Fig. 2:**
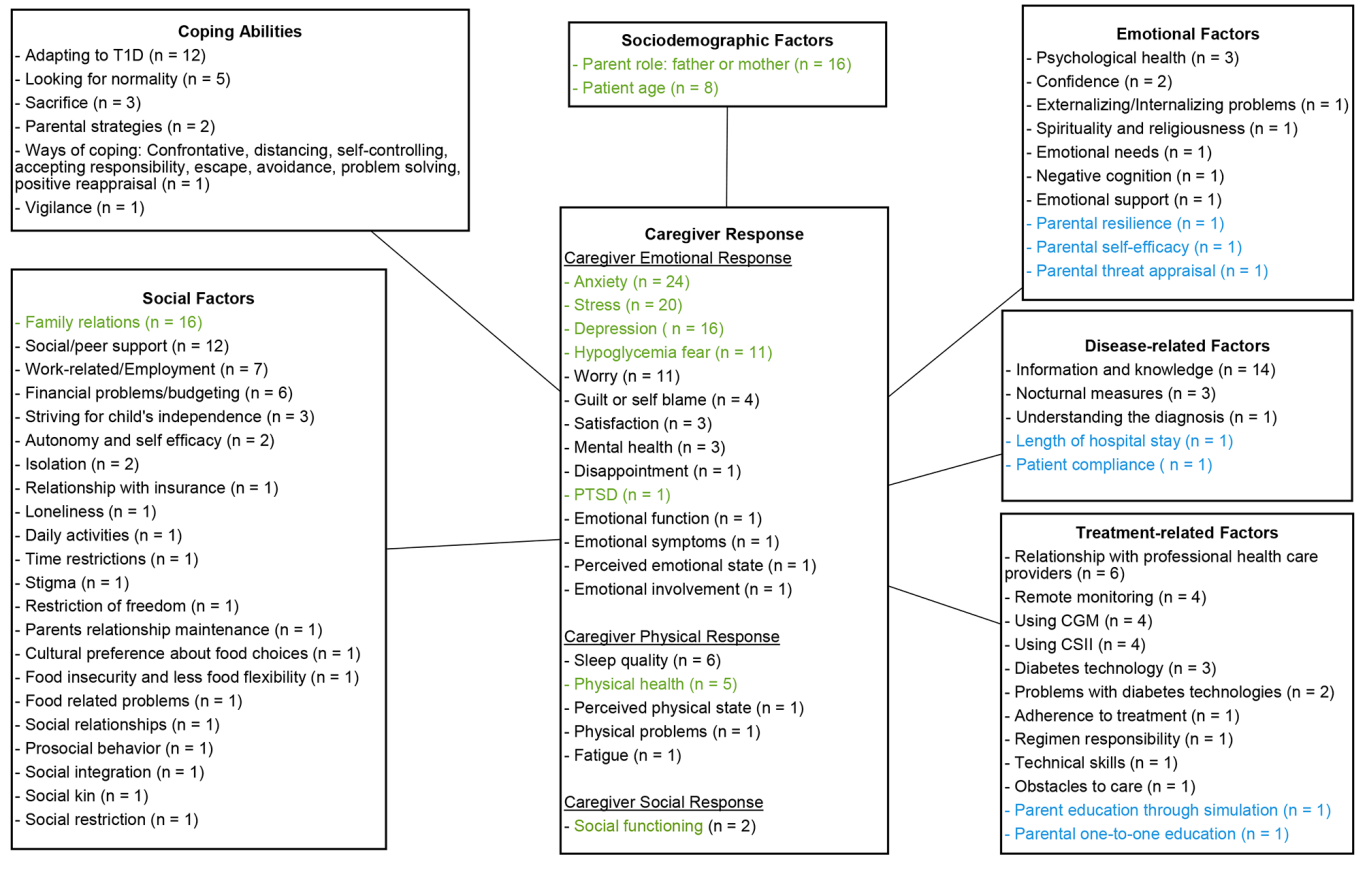
Literature-based conceptual framework on burden of pediatric T1D on informal caregivers Elements in blue-colored font were found only in caregivers of children who were recently diagnosed with T1D; elements in black-colored font were found only in caregivers of children with T1D for at least three months; elements in green-colored font were found in caregivers of children with T1D of any duration (both recently diagnosed and after three months of diagnosis)

The review of the literature has identified a series of factors that affect the QoL of informal caregivers of children with T1D, with a direct impact on physical, emotional, and social outcomes. The challenges identified for the caregivers of children with T1D were associated with a certain number of factors that have been classified into six different categories (Fig. 2): (i) Category of social factors such as social support, work-related problems, financial burden, striving for child’s independence, and isolation; (ii) Coping abilities-related factors included adapting to T1D, looking for normality, sacrifice, adaptation process to T1D, and parental strategies; (iii) the category of emotional factors included psychological health and self-confidence; (iv) Disease-related factors such as information about the disease and nocturnal measures also impacted the burden of caregivers; (v) Socio-demographic category included the parental role and patient age; and (vi) the category of treatment-related factors included relationship with professional health-care providers and using remote monitoring and diabetes technologies.

These six categories of factors have an impact on the caregiver’s social, physical, and emotional outcomes in the context of T1D. The way caregivers respond to burden also varied according to the duration since diagnosis. Caregivers experience more anxiety, stress, depression, fear of hypoglycemia, physical health problems, and problems with social functioning regardless of whether their child was recently diagnosed, indicating that potentially these impacts could be experienced throughout the caregiver journey with T1D. Additionally, caregivers of recently diagnosed children with T1D further experience worriedness, guilt, self-blame, dissatisfaction, mental distress, and a decrease in sleep quality. An overview of the literature-based conceptual framework, including the number of reporting studies per element, is depicted in Fig. 2.

### Common burden related to diagnosis of T1D, shared by children and informal caregivers as part of the family unit

**Fig. 3:**
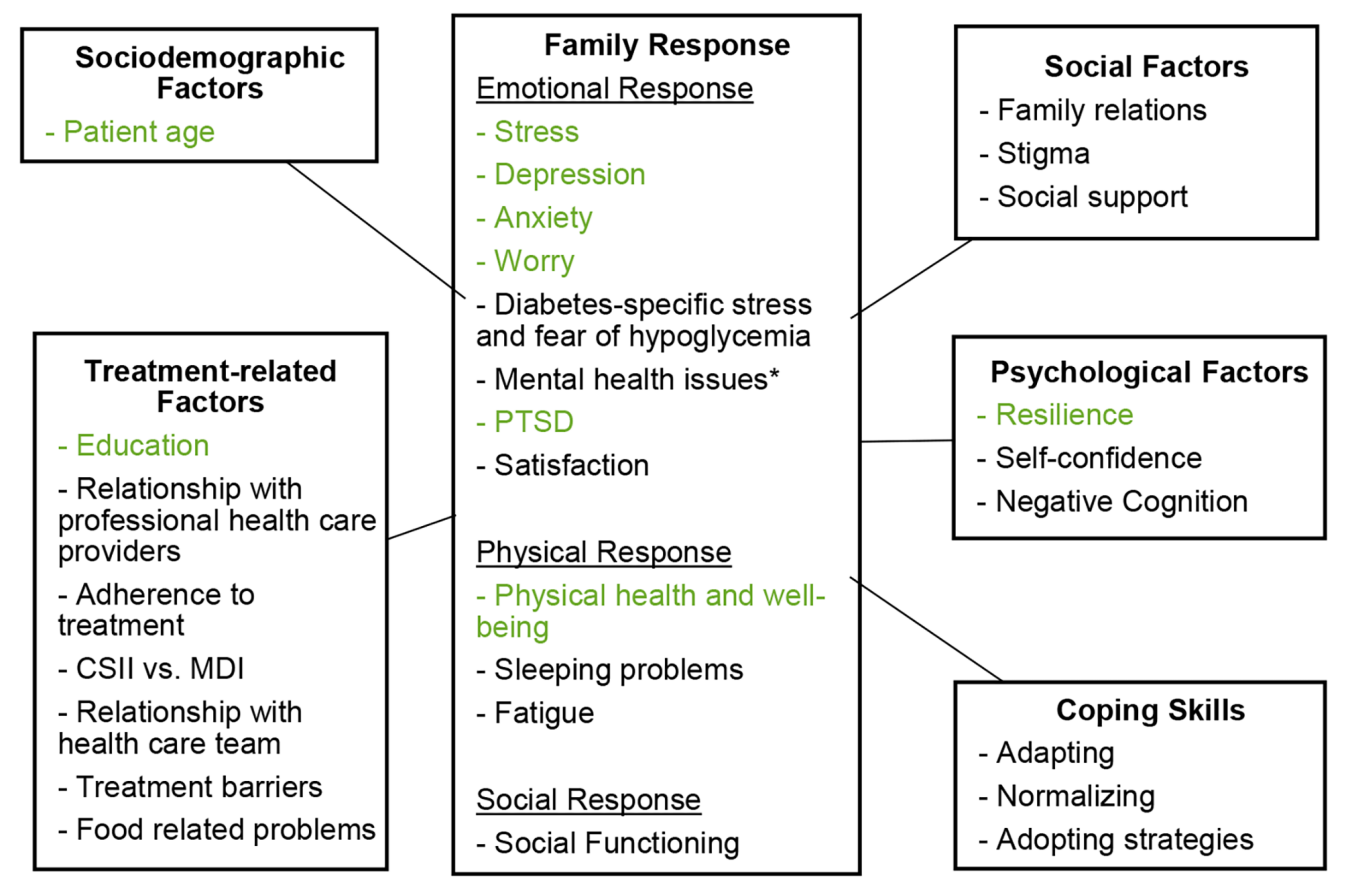
Literature-based conceptual framework on burden of pediatric T1D on children and informal caregivers as part of the family unit *Mental health was measured using SF-12 CSII: Continuous Subcutaneous Insulin Infusion; MDI: Multiple daily injections Elements in black-colored font were found only in children with T1D for at least three months and their caregivers; elements in green-colored font were found in children with T1D of any duration (both recently diagnosed and after three months of diagnosis) and their caregivers

Based on the four SLRs on the burden of T1D in children and caregivers, we described the burden of T1D that is common for both children and caregivers as part of a family unit. These common challenges, presented in Figs. 1 and 2, are unique, as they develop in the context of a dynamic relationship between child and caregiver, which makes them amenable to specific interventions using the transactional interaction between members of a family. The conceptual framework showing the common burden on children and caregivers, further categorized into mental, emotional, and physical burden, and the related factors, is presented in Fig. 3.

Factors associated with the development of a common burden have been grouped into 5 categories: socio-demographic factors, treatment-related factors, social factors, psychological factors, and coping skills. Children’s age affects how both caregivers and children experience burden, shortly after diagnosis and any time after. The category of treatment-related factors includes education on the treatment method shortly after diagnosis, while the relationship with the team of healthcare providers, treatment adherence, the insulin administration method, barriers to the treatment, and dietary problems were reported in studies looking at the consequences of T1D any time after diagnosis. Family support and social support as well as stigma affected the common burden regardless of the disease duration. Emotional factors include resilience, which affected children and caregivers in both recent diagnosis of T1D and after, while self-confidence and negative cognition were reported in the context of a recent diagnosis only. Additionally, common coping factors include adapting, normalizing, and adopting strategies (Fig. 3).

Children and caregivers showed similar patterns of responses to disease. On the emotional level, both children and caregivers experience stress, depression, anxiety, and worry, which were reported in studies looking at recently diagnosed T1D as well as any duration of T1D. Mental health issues are also a common burden for children and caregivers, after a longer period of diagnosis. On the physical level, both caregivers and children report problems with their physical well-being immediately after the diagnosis and later. Sleeping problems and fatigue appeared in studies reporting on patients with at least three months of T1D. Finally, caregivers and children report a burden on their social functioning, which was only reported in studies focusing on patients being diagnosed for longer than three months.

## Discussion

This series of SLRs described and characterized the landscape of evidence on the humanistic burden of children with T1D and their informal caregivers. Three literature-based conceptual frameworks were built to identify and evaluate the impact of diagnosis of pediatric T1D on children and their caregivers and the common burden children and caregivers share in the same family.

The time of diagnosis was taken into consideration, as the onset of the disease is often an unexpected and shocking event for both children and caregivers and may cause acute effects, while the burden at a later period is largely chronic in nature. We found that more studies were conducted to identify the burden of established T1D as opposed to newly diagnosed, which was the main reason for finding fewer factors of burden in the context of a recent T1D diagnosis. Additionally, most of the reported associations are simple correlations based on cross-sectional studies with limited causal inference, as opposed to relationships observed in cohort studies, which are less prone to biases and a stronger study design to establish causal relationships. Nonetheless, most of these associations were expected, and considered a priori in the original protocol; therefore, they represent useful ‘indicators’ to trigger attention for future interventions and possibly serve as ‘outcome markers’ of psycho-social-educative interventions and possibly drug interventions.

We identified a certain number of specific challenges that both the child with T1D and the caregiver experienced. Anxiety, depression, stress, and worry were commonly found among children and caregivers facing a recent diagnosis of T1D and continued over time in relation to new challenges pertaining to aging or disease complexity.

Generally, a child’s age was related to the burden on both the child and caregiver with different expressions at different ages. Parents of adolescents experienced higher levels of stress than parents of younger children. At the same time, the child’s age was shown to be an influencing factor that affected QoL. Adolescents showed worse emotional response and school functioning, more disturbed QoL, and higher levels of depression when compared with younger patients [17–26]. Patients in the later stages of adolescence seemed to have the worst depression and anxiety symptoms. This is concordant with the findings of Lasaite 2016, which concluded that patients with T1D displayed a greater burden of diabetes distress in emerging adulthood than in adolescence [27]. These findings were expected, as both children and parents will have more burden when children grow older and become independent and gain responsibility for their treatment. However, some other factors could have played a role; for example, the limitations of the scales used to assess depression in young children, and the fact that younger patients may not describe their feelings as well as older ones.

To our surprise, symptoms like fatigue, sleeping problems, mental health, and social functioning were reported among both the child and caregiver, only in populations with a diagnosis of T1D for longer than 3 months. This association may reflect the fact that the newly diagnosed population is not very well studied, or children/caregivers have different important priorities immediately after the T1D diagnosis where the attention is focused on learning how to cope with the new disease. Another interpretation, however, is that the level of support at the time of diagnosis is efficient and focused on decreasing stress and anxiety.

Finally, we may also consider the need to have enough experience with the disease and its treatments to realize the complexity and daily life challenges. Worriedness can be justified in the first three months after diagnosis of T1D by the fear of acute and long-term complications and pressure to achieve “treatment goals”. At another stage, the impact of disease on the child’s life, in general, becomes a major concern with chronic consequences, as illustrated by the relationship between the occurrence of T1D before 10 years of age and the loss of 17.7 life years among women and 14.2 life years in men [28].

**Fig. 4:**
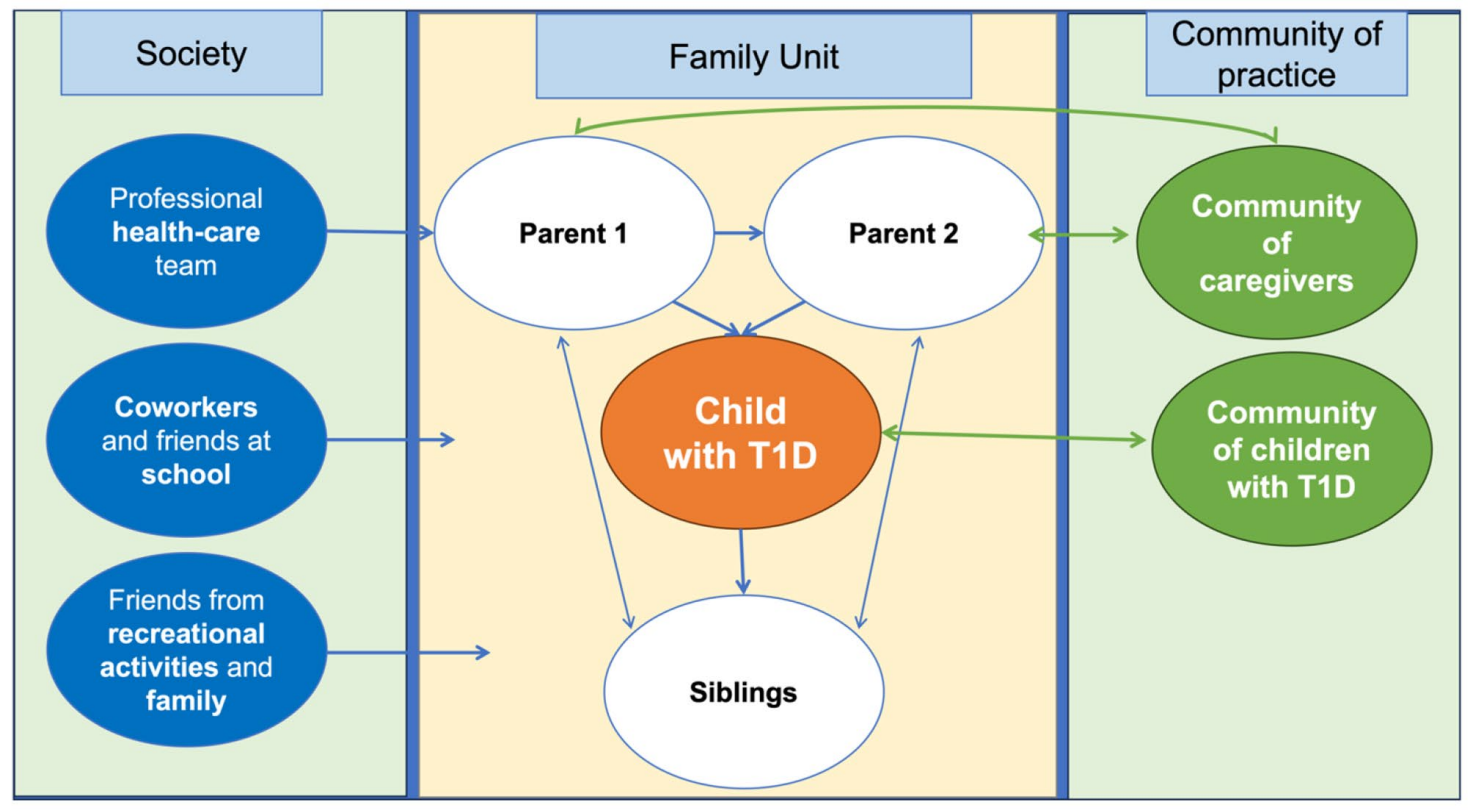
Diagram of transactional family dynamics and external supports for children with T1D and caregivers Legend: Central part of the figure shows the transactional dynamics within a family with a child with T1D, independent of the living arrangements of the family. These interactions are between the parents, between each parent and each child (including the child with T1D), and among siblings including interactions with the child who has T1D. These dynamics are also affected by external factors: (i) spontaneous social interactions with other family members, extended family, and professional relationships as well as school friends and teachers for the child. (ii) Another form of support is through educational interactions with the healthcare team that can influence the way children and caregivers react to the disease and use treatment technologies. (iii) Finally, the family dynamic is also influenced by the participation of the child with T1D and the caregiver to specific communities of practice where they can meet peers to exchange experiences and ask questions. Once quality of life, resilience and mental health improve in one family member, this leads to an improvement in other family members. Figure 4 describes this dynamic

The psychological and emotional burden experienced by both children and caregivers was expected because family members tend to influence one another emotionally when they share a household, through transactional interaction (Fig. 4). Family dynamics include complex patterns of influence in interparental, father-child, mother-child, and sibling relationships. Within a family, children are not passive recipients of parenting, but rather, active participants in parent-child relationships, and therefore, the frameworks are bi-directional and multi-faceted [29]. Parenting practices and child functioning are a product of both parent and child characteristics and behavior. Parent-child interactions occur in a wide range of contexts (e.g., playing, caregiving, teaching, daily life activities), and parent-child interactions in one context may affect interactions in another context. Moreover, the relationship between parents and children is affected by the intentional influence on life circumstances and exercising the ability to engage in intentional behavior [8, 30, 31]. In the context of the family unit, even small events can trigger emotional reactions or anxiety, which may influence other family members. The opposite is also true, where a mutual family experience (for instance, successful support networking) can lead to changes in one individual [30] Moment-by-moment influence processes contribute to long-term influence processes, as well as the reverse, and both long and short periods of time uniquely contribute to the whole of family experience [30, 31]. Another point to be taken into consideration when analyzing the conceptual frameworks is that an individual is part of a whole, and that to understand the individual, one must examine the interrelations between one part and the whole. This transactional concept exists beyond the family [31] when children interact with peers at school, while parents also function in their work environments (Fig. 4) [30].

The burden that children with T1D and their caregivers experience is related to the specific way children and caregivers experience the new disease and its management. The nature of the elements that generate burden is different for the children and the caregivers. For instance, children reported burden due to T1D symptoms, blood sugar testing frequency, duration of the disease, and failing with the disease routine or not reaching treatment targets. Meanwhile, caregivers reported more practical factors related to diabetes management, such as a lack of information and understanding about the diagnosis, the relationship with the healthcare team, and nocturnal measuring. Additionally, treatment-related factors that affected both children and caregivers included treatment adherence: caregivers reported challenges regarding diabetes technology and skills, while children reported having difficulty with adopting new practices or behaviors as a main source of difficulty. Furthermore, emotionally, children showed signs of sadness, discouragement, and embarrassment, while parents showed disappointment and anxiety as an emotional response. On the social response level, children experienced more challenges than their parents, including loneliness and social withdrawal, as well as social stress (Tables 1 and 2).

Finally, we identified factors that play a role in the way that T1D affects children and their caregivers. For instance, female patients had more disturbed QoL, and worse symptoms of depression and anxiety compared to male patients. This is similar to the general adolescent population, where girls tend to have more mental health challenges and girls’ QoL declines more than boys over time [32]. The risk for mental health problems increases among women when they transition to adulthood knowing that women’s status and life opportunities remain low worldwide [33]. Similarly, among caregivers, mothers experience higher levels of burden when compared to fathers. Mothers generally have higher levels of anxiety and fear of hypoglycemia than fathers. Mothers also expressed more problems related to the future of their children’s T1D, including treatment, and the long-term consequences.

Since emotional and mental health problems can be affected by other family members (Fig. 4), the development of resilience and self-efficacy would follow the same pattern. And so, if one family member gains confidence or has improved mental health, other members of the family will benefit from these improvements [34]. Group interventions to support children with T1D and caregivers through specific networks are useful interventions to learn in action, share information and resources, and support each other. Within these communities of patients or caregivers, some educational programs can be proposed to control stress and promote resilience to help individuals and families with similar experiences. Families can work together to support each other to share new knowledge and skills toward developing self-efficacy and self-confidence [35]. As illustrated in Fig. 4, these different interventions strengthen the participating child or caregiver, which has a positive impact on the other. The process makes the whole family stronger and helps children with T1D to progressively incorporate the diagnosis of the disease into a new identity (i.e., “children with diabetes”), which in turn improves clinical outcomes [36].

At the same time, progress in technology and therapeutics in T1D is offering new opportunities to improve QoL, reduce the burden of disease, and improve understanding of T1D and its treatment. For example, the use of an Automated Insulin Delivery system in children with T1D was associated with an improvement in psychosocial outcomes in children and their caregivers [37]. Evaluation of the burden on children with T1D as well as their families should become a standard in conducting interventional trials of new devices and new treatments. More research is needed to understand better the time dynamics of the burden from a period close to diagnosis to the time when the disease is established.

This manuscript is based on four SLRs covering the burden of pediatric T1D on children and their informal caregivers. The SLRs covered nested timelines, which was important to describe the specific effect of the diagnosis, as well as the effect over a longer duration of disease. To our knowledge, this is the first approach to understand both the specific burden of T1D on children and caregivers, as well as the common burden.

However, this review has limitations. Only a limited number of studies reported the burden of T1D on children and caregivers within the first few months of diagnosis. We also observed a large heterogeneity among the studies regarding the instruments used and the quality of the reporting (see Additional File 4 for a summary of the quality assessment). Finally, most of the included studies in this review were conducted in developed countries, which may limit the inference to other countries or populations that live with different health/education systems, priorities, cultures, and preference. Caregiving practices vary significantly across cultures, shaped by diverse social norms, beliefs, and values [38]. In some cultures, caregiving is deeply ingrained in familial roles and responsibilities, with extended family members often sharing caregiving duties. In contrast, individualistic cultures may prioritize autonomy and independence, leading to different approaches to caregiving. Additionally, cultural beliefs about illness and quality of life can shape perceptions of caregiving, impacting the level of support and resources available. These cultural differences can influence how individuals perceive the burden of T1D. Understanding these cultural differences is essential for providing culturally sensitive and effective support to families across diverse backgrounds. Despite these limitations, these results invite others to conduct more research to better understand the humanistic burden of T1D among children with T1D and their caregivers, especially during the period that follows the diagnosis.

Overall, this study shows that the burden of T1D extends beyond the individual diagnosed and encompasses informal caregivers within the family unit. This shared burden underscores the necessity for interventions tailored to address the dynamic interaction between children and caregivers. Comprehensive support strategies must consider various contributing factors, including socio-demographic, treatment-related, social, psychological, and coping aspects. Additionally, interventions should be tailored according to the age of the child to effectively mitigate the burden experienced. Despite varying durations of T1D, both children and caregivers exhibit consistent patterns of emotional and physical burden, emphasizing the need for holistic approaches that promote family resilience and provide age-appropriate support. By addressing the shared burden within the family unit, interventions can enhance coping skills, provide education, and facilitate social support, ultimately improving outcomes for both children and family members affected by T1D.

## Conclusions

T1D causes a significant burden to affected children and their caregivers, both independently and through transactional interaction within the family unit. One type of strategy to reduce the disease burden is to strengthen individuals for the benefit of the whole family using the same transactional interaction. The review sheds light on the various aspects of burden experienced by children with T1D and their caregivers, including anxiety, depression, stress, and worriedness. Additionally, information from this review could be used to support children and caregivers either to prevent the development of excessive burden or to mitigate its development.

This work identified significant gaps in the evidence base regarding disease burden in children who are newly diagnosed with T1D and their informal caregivers. People with newly diagnosed T1D and their informal caregivers form an underserved and under-researched population. The scarcity of evidence and limited quality of the few included studies draw attention to some of the key areas of unmet needs for this population. Overall, this manuscript underscores the need for continued research including longitudinal studies and prospective interventional trials to better understand the effects of different approaches on the burden on families living with a child with T1D.

### Electronic supplementary material

Below is the link to the electronic supplementary material.

Additional File 1: PICO eligibility criteria (Overview of PICO eligibility criteria that were used for study selection in the systematic literature reviews)

Additional File 2: Search strategies (Search strategies for database and gray literature searches that were conducted for the systematic literature reviews)

Additional File 3: Study selection (PRISMA flow diagrams and lists of included studies from the systematic literature reviews)

Additional File 4: Quality assessment (Summary of quality assessment of included studies from the systematic literature reviews)

## Data Availability

The data supporting this systematic review are from previously reported studies and datasets, which have been cited.

## Abbreviations

CGM: Continuous glucose monitoring
CSII: Continuous subcutaneous insulin infusion
MDI: Multiple daily injections
PICO: Population, Intervention, Comparator, and Outcome
PRISMA: Preferred Reporting Items for Systematic Reviews and Meta-Analyses
QoL: Quality of life
SLR: Systematic literature review
T1D: Type 1 diabetes
