# Supplementary material for: Humanistic burden of pediatric type 1 diabetes on children and informal caregivers: systematic literature reviews
Source: Diabetol Metab Syndr. 2024 Mar 21;16:73. doi: 10.1186/s13098-024-01310-2 (PMC10956250; doi:10.1186/s13098-024-01310-2)
Supplement: Supplementary file 1 — Additional File 1: PICO eligibility criteria (Overview of PICO eligibility criteria that were used for study selection in the systematic literature reviews) [file 13098_2024_1310_MOESM1_ESM.docx]

# Additional File 1: PICO eligibility criteria

**Table A1.1: PICO eligibility criteria** **for patients newly diagnosed with T1D SLR**

| **PICO item** | **Inclusion criteria** | **Exclusion criteria** |
| --- | --- | --- |
| **Population** | Pediatric/adolescent/young adult patients (aged 6 to 21) with newly/recently diagnosed type 1 diabetes (within the last 3 months) | N/A |
| **Intervention/ Comparator** | Any or none | N/A |
| **Outcomes** | - Disease/symptom impact on patients - Outcomes regarding patient perspective, symptoms, and concepts of importance using generic or disease-specific scales or patient-reported outcomes, including but not limited to:   - PROMIS® pediatric self-report scales   - Pediatric Quality of Life Inventory (PedsQL)   - Child health questionnaire (CHQ)   - DISABKIDS Chronic Generic Measure (DCGM)   - Revised Health-Related Quality of Life Questionnaire for Children and Adolescents (KINDL-R)   - Quality of My Life Questionnaire (QoML)   - Adolescent coping orientation for problem experiences scale (ACOPE)   - Adolescent quality of life scale (AQOL)   - Beck depression inventory (BDI)   - Children’s depression inventory (CDI)   - Children’s negative cognitive error questionnaire (CNCEQ)   - Illness perception questionnaire (IPQ)   - Life stressors and social resources inventory (LISRES-Y)   - State anxiety inventory for children (SAIC)   - Summary of self-care activities scale (SSCAS)   - Diabetes stress questionnaire (DSQ)   - Diabetes compliance questionnaire (DCQ)   - Diabetes distress scale (DDS)   - Diabetes independence survey (DIS)   - Diabetes quality of life for youth scale (DQOLY)   - Issues in coping with diabetes scale–Upset subscale (ICD)   - Problem areas in diabetes scale (PAID)   - Personal models of diabetes questionnaire (PMD)   - Self efficacy for diabetes scale (SED)   - EuroQol-5 Dimension (EQ-5D)   - EuroQol-Visual Analogue Scale (EQ-VAS)   - 36-Item Short Form Survey (SF-36)   - Satisfaction with Life Scale (SLS)   - Hospital Anxiety and Depression Scale (HADS)   - Quality of Well-Being Scale (QWB)   - Feelings or experience after diagnosis (e.g., denial, shock)   - Feelings about burdening a caregiver due to the disease (e.g., worry, guilt)   - Treatment satisfaction | N/A |
| **Study design** | - Randomized controlled trials - Non-randomized clinical trials - Observational evidence (including cohort studies, case-control, and cross-sectional studies) - Qualitative research (e.g., focus groups, interviews) - Letters   **For library:**   - Systematic literature reviews, with or without meta-analyses | - Narrative reviews - Guidelines - Case reports/series - Editorials and Notes |
| **Additional criteria (limits)** | | |
| **Language** | English only | |

N/A, not applicable.

**Table A1.2: PICO eligibility criteria for patients diagnosed with T1D of any duration SLR**

| **PICO item** | **Inclusion criteria** | **Exclusion criteria** |
| --- | --- | --- |
| **Population** | Children/adolescents/young adults (aged 6 to 21) with type 1 diabetes of any duration | N/A |
| **Intervention/ Comparator** | Any or none | N/A |
| **Outcomes** | - Disease/symptom impact on patients - Outcomes regarding patient perspective, symptoms, and concepts of importance using generic or disease-specific scales or patient-reported outcomes, including but not limited to:   - PROMIS® pediatric self-report scales   - Pediatric Quality of Life Inventory (PedsQL)   - Child health questionnaire (CHQ)   - DISABKIDS Chronic Generic Measure (DCGM)   - Revised Health-Related Quality of Life Questionnaire for Children and Adolescents (KINDL-R)   - Quality of My Life Questionnaire (QoML)   - Adolescent coping orientation for problem experiences scale (ACOPE)   - Adolescent quality of life scale (AQOL)   - Beck depression inventory (BDI)   - Children’s depression inventory (CDI)   - Children’s negative cognitive error questionnaire (CNCEQ)   - Illness perception questionnaire (IPQ)   - Life stressors and social resources inventory (LISRES-Y)   - State anxiety inventory for children (SAIC)   - Summary of self-care activities scale (SSCAS)   - Diabetes stress questionnaire (DSQ)   - Diabetes compliance questionnaire (DCQ)   - Diabetes distress scale (DDS)   - Diabetes independence survey (DIS)   - Diabetes quality of life for youth scale (DQOLY)   - Issues in coping with diabetes scale–Upset subscale (ICD)   - Problem areas in diabetes scale (PAID)   - Personal models of diabetes questionnaire (PMD)   - Self-efficacy for diabetes scale (SED)   - EuroQol-5 Dimension (EQ-5D)   - EuroQol-Visual Analogue Scale (EQ-VAS)   - 36-Item Short Form Survey (SF-36)   - Satisfaction with Life Scale (SLS)   - Hospital Anxiety and Depression Scale (HADS)   - Quality of Well-Being Scale (QWB)   - Feelings or experience after diagnosis (e.g., denial, shock)   - Feelings about burdening a caregiver due to the disease (e.g., worry, guilt)   - Treatment satisfaction | N/A |
| **Study design** | - Randomized controlled trials (RCT) - Non-randomized clinical trials - Observational evidence (including cohort studies, case-control, and cross-sectional studies) - Qualitative research (e.g., focus groups, interviews) - Letters   **For library:**   - Systematic literature reviews, with or without meta-analyses   **For summary of intervention characteristics:**   - Systematic literature reviews, with or without meta-analyses discussing interventions in the context of type 1 diabetes | - Narrative reviews - Guidelines - Case reports/series - Editorials and Notes |
| **Additional criteria (limits)** | | |
| **Language** | English only | |

N/A, not applicable.

**Table A1.3: PICO eligibility criteria for caregivers of patients newly diagnosed with T1D SLR**

| **PICO item** | **Inclusion criteria** | **Exclusion criteria** |
| --- | --- | --- |
| **Population** | Informal caregivers or parents of children and young adults (ages 6 to 21), who have been newly diagnosed with T1D within approximately three months (including type 1a and type 1b) | - Formal caregivers (e.g., doctors, nurses, and other healthcare professionals) - Studies including mixed diagnoses were excluded, unless subgroup data was reported for T1D caregivers or care partners |
| **Interventions/ Comparators** | Any or none | N/A |
| **Outcomes** | Patient disease/symptom impact on caregiver/care partner  Outcomes regarding informal caregiver/care partner perspective, symptoms, and concepts of importance, included but not limited to:   - Family Adaptation and Cohesion Evaluation Scale (FACES) - Days absent from work (e.g., absenteeism and presenteeism) - Hospital anxiety and depression scale (HADS) - Zarit Burden Scale - CareQoL - Caregiver Reaction Assessment (CRA) - Kingston Caregiver Stress Scale (KCSS) - Caregiver burden inventory - Caregiver strain index - Burden scale for family caregivers - Caregiver burden scale - Folkman’s 4-item measure of finding positive meaning in caregiving - Health-related quality of life (HRQoL) - Beck Depression Inventory; Beck Hopelessness Scale - Chalder Fatigue Scale - Physical Fatigue subscale - 6-item Short Form Health Survey Questionnaire - 6-item Short Form Health Survey Questionnaire Mental Component - 36-item Short Form Health Survey Questionnaire Physical Component - Zung Depression Scale - State-Trait Anxiety Inventory - Utrecht Coping List Passive Approach subscale   Objective outcomes:   - Missed days at work in the past year due to caregiving responsibilities - Loss of job/employment - Lost income - Sleep quality/loss of sleep | Outcomes that did not directly measure or provide insight to caregiver/care partner burden |
| **Study design** | - Randomized controlled trials - Non-randomized clinical trials - Observational studies (including cohort studies, case-control, and cross-sectional studies) - Qualitative research (e.g., focus groups, interviews)   For library:   - Systematic literature reviews, with or without meta-analyses | - Narrative reviews - Guidelines - Case reports/series - Editorials, Notes, and Letters |
| **Additional criteria (limits)** | | |
| **Language** | English only | |

N/A: Not applicable.

**Table A1.4: PICO eligibility criteria for caregivers of patients with T1D of any duration SLR**

| **PICO item** | **Inclusion criteria** | **Exclusion criteria** |
| --- | --- | --- |
| **Population** | Informal caregivers or parents of children and young adults (ages 6 to 21) with T1D of any duration (including type 1a and type 1b) | - Formal caregivers - Studies including mixed diagnoses, unless subgroup data is reported for T1D caregivers or care partners |
| **Interventions/ Comparators** | Any or none | N/A |
| **Outcomes** | Patient disease/symptom impact on caregiver/care partner  Outcomes regarding informal caregiver perspective, symptoms, and concepts of importance, including but not limited to:   - Family Adaptation and Cohesion Evaluation Scale (FACES) - Days absent from work (e.g., absenteeism and presenteeism) - Hospital anxiety and depression scale (HADS) - Zarit Burden Scale - CareQoL - Caregiver Reaction Assessment (CRA) - Kingston Caregiver Stress Scale (KCSS) - Caregiver burden inventory - Caregiver strain index - Burden scale for family caregivers - Caregiver burden scale - Folkman’s 4-item measure of finding positive meaning in caregiving - Health-related quality of life (HRQoL) - Beck Depression Inventory; Beck Hopelessness Scale - Chalder Fatigue Scale - Physical Fatigue subscale - 6-item Short Form Health Survey Questionnaire - 6-item Short Form Health Survey Questionnaire Mental Component - 36-item Short Form Health Survey Questionnaire Physical Component - Zung Depression Scale - State-Trait Anxiety Inventory - Utrecht Coping List Passive Approach subscale   Objective outcomes:   - Missed days at work in the past year due to caregiving responsibilities - Loss of job/employment - Lost income - Sleep quality/loss of sleep | Outcomes not directedly measuring or providing insight to caregiver burden |
| **Study design** | - Randomized controlled trials - Non-randomized clinical trials - Observational studies (including cohort studies, case-control, and cross-sectional studies) - Qualitative research (e.g., focus groups, interviews)   For library:   - Systematic literature reviews, with or without meta-analyses | - Narrative reviews - Guidelines - Case reports/series - Editorials, Notes, and Letters |
| **Additional criteria (limits)** | | |
| **Language** | English only | |

N/A: Not Applicable
