# Supplementary material for: Humanistic burden of pediatric type 1 diabetes on children and informal caregivers: systematic literature reviews
Source: Diabetol Metab Syndr. 2024 Mar 21;16:73. doi: 10.1186/s13098-024-01310-2 (PMC10956250; doi:10.1186/s13098-024-01310-2)
Supplement: Supplementary file 2 — Additional File 2: Search strategies (Search strategies for database and gray literature searches that were conducted for the systematic literature reviews) [file 13098_2024_1310_MOESM2_ESM.docx]

# Additional File 2: Search strategies

**Search strategy for patients newly diagnosed with T1D SLR**

**Table A2.1**: Search strategy for Embase via OvidSP

| **Database: Embase 1974 to March 22, 2022**  **Search executed: March 23, 2022** | | |
| --- | --- | --- |
| **#** | **String** | **Hits** |
| 1 | exp insulin dependent diabetes mellitus/ | 127729 |
| 2 | (((Type* adj3 "1") or "I" or one*) adj3 (diabete* or diabetic*)).ti,ab. | 111374 |
| 3 | (insulin* adj3 depend* adj3 (diabete* or diabetic*)).ti,ab. | 31561 |
| 4 | (type 1 diabete$ or type 1 diabetic* or diabetes mellitus, type 1 or juvenile diabete$ or insulin dependent diabete$ mellitus).ti,ab. | 95785 |
| 5 | or/1-4 | 178953 |
| 6 | socioeconomics/ | 151415 |
| 7 | exp Quality of Life/ | 572480 |
| 8 | quality of life.ti,kw. | 155198 |
| 9 | ((instrument or instruments) adj3 quality of life).ab. | 5088 |
| 10 | (sf36 or sf 36 or short form 36 or shortform 36 or short form36 or shortform36 or sf thirtysix or sfthirtysix or sfthirty six or sf thirty six or shortform thirtysix or shortform thirty six or short form thirtysix or short form thirty six).ti,ab,kw. | 46285 |
| 11 | (sf6 or sf 6 or short form 6 or shortform 6 or sf six or sfsix or shortform six or short form six or shortform6 or short form6).ti,ab,kw. | 2700 |
| 12 | (sf8 or sf 8 or sf eight or sfeight or shortform 8 or shortform 8 or shortform8 or short form8 or shortform eight or short form eight).ti,ab,kw. | 949 |
| 13 | (sf12 or sf 12 or short form 12 or shortform 12 or short form12 or shortform12 or sf twelve or sftwelve or shortform twelve or short form twelve).ti,ab,kw. | 10982 |
| 14 | (sf16 or sf 16 or short form 16 or shortform 16 or short form16 or shortform16 or sf sixteen or sfsixteen or shortform sixteen or short form sixteen).ti,ab,kw. | 64 |
| 15 | (sf20 or sf 20 or short form 20 or shortform 20 or short form20 or shortform20 or sf twenty or sftwenty or shortform twenty or short form twenty).ti,ab,kw. | 489 |
| 16 | (hql or hqol or h qol or hrqol or hr qol).ti,ab,kw. | 34134 |
| 17 | (pqol or qls).ti,ab,kw. | 694 |
| 18 | (quality of wellbeing or quality of well being or index of wellbeing or index of well being or qwb).ti,ab,kw. | 811 |
| 19 | nottingham health profile*.ti,ab,kw. | 1615 |
| 20 | nottingham health profile/ | 580 |
| 21 | sickness impact profile.ti,ab,kw. | 1268 |
| 22 | sickness impact profile/ | 2361 |
| 23 | (eq or euroqol or euro qol or eq5d or eq 5d or euroqual or euro qual).ti,ab,kw. | 32735 |
| 24 | duke health profile.ti,ab,kw. | 115 |
| 25 | functional status questionnaire.ti,ab,kw. | 165 |
| 26 | dartmouth coop functional health assessment*.ti,ab,kw. | 13 |
| 27 | (PROMIS or "Patient-Reported Outcomes Measurement Information System").ti,ab. | 6245 |
| 28 | Visual Analog Scale/ | 104864 |
| 29 | (visual analogue scale or visual analog scale).ti,ab,kw. | 82693 |
| 30 | exp patient-reported outcome/ | 38891 |
| 31 | ("patient reported" or "patient-reported").ti,ab. | 78104 |
| 32 | (humanistic adj3 (burden or impact or outcome)).ti,ab. | 874 |
| 33 | ((Disability and Impact Profile) or Disability Impact Profile).ti,ab,kw. | 601 |
| 34 | ("Performance Scale*" or "treatment satisfaction" or "Computerized Adaptive Testing" or "Empowerment Scale" or "impact survey").ti,ab. | 11126 |
| 35 | (Beck Depression Inventory or Appraisal of Diabetes Scale or Diabetes 39 or Audit of Diabetes-Dependent Quality of Life or ADDQoL or Diabetes Quality of Life Measure or DQoL or eDiabetes Health Profile or Diabetes Quality of Life Clinical Trial Questionnaire or Barriers to Physical Activity in Diabetes or Diabetes Obstacles Questionnaire or Diabetes Treatment Satisfaction Questionnaire or DTSQ or Diabetes Treatment Satisfaction Questionnaire for Inpatients or Diabetes Symptom Checklist-Revised or Diabetes Computerized Adaptive Testing or Diabetes Impact Survey or Insulin Treatment Satisfaction Questionnaire or Diabetes Empowerment Scale or Diabetes Specific Quality of Life Questionnaire or Satisfaction with Oral Anti-Diabetic Agent Scale).ti,ab. | 22295 |
| 36 | or/6-35 | 946347 |
| 37 | 5 and 36 | 8045 |
| 38 | (exp animal/ or nonhuman/) not exp human/ | 6770592 |
| 39 | (book or chapter or editorial or erratum or note or short survey or tombstone or comment).pt. | 2275636 |
| 40 | Case Study/ | 84432 |
| 41 | case report.tw. | 480140 |
| 42 | or/38-41 | 9487102 |
| 43 | (conference or conference abstract or conference review).pt. | 5127456 |
| 44 | limit 43 to yr="2020 -Current" | 553563 |
| 45 | 43 not 44 | 4573893 |
| 46 | 42 or 45 | 13410377 |
| 47 | 37 not 46 | 5586 |
| 48 | ("paediatric*" or "pediatric*" or "child*" or "kid*" or "young adult*" or "teen*" or "adolescen*" or "boy*" or "girl*" or "youngster*" or "juvenil*" or "minor*" or "boyhood" or "schoolchild*" or "school child*" or "youth*" or "under*age" or "pubescen*" or "school*" or "prematur*" or "preterm*").ti,ab. | 4132024 |
| 49 | exp child/ | 2858586 |
| 50 | 48 or 49 | 5186129 |
| 51 | 47 and 50 | 2124 |

**Table A2.2**: Search strategy for MEDLINE® via OvidSP

| **Database: MEDLINE 1946 to March 22, 2022**  **Search executed: March 23, 2022** | | |
| --- | --- | --- |
| **#** | **String** | **Hits** |
| 1 | exp Diabetes Mellitus, Type 1/ | 82163 |
| 2 | (((Type* adj3 "1") or "I" or one*) adj3 (diabete* or diabetic*)).ti,ab. | 71497 |
| 3 | (insulin* adj3 depend* adj3 (diabete* or diabetic*)).ti,ab. | 26770 |
| 4 | (type 1 diabete$ or type 1 diabetic* or diabetes mellitus, type 1 or juvenile diabete$ or insulin dependent diabete$ mellitus).ti,ab. | 65237 |
| 5 | or/1-4 | 123895 |
| 6 | Socioeconomic Factors/ | 168487 |
| 7 | exp "Quality of Life"/ | 235979 |
| 8 | quality of life.ti,kw. | 98434 |
| 9 | ((instrument or instruments) adj3 quality of life).ab. | 3698 |
| 10 | (sf36 or sf 36 or short form 36 or shortform 36 or short form36 or shortform36 or sf thirtysix or sfthirtysix or sfthirty six or sf thirty six or shortform thirtysix or shortform thirty six or short form thirtysix or short form thirty six).ti,ab,kw. | 28517 |
| 11 | (sf6 or sf 6 or short form 6 or shortform 6 or sf six or sfsix or shortform six or short form six or shortform6 or short form6).ti,ab,kw. | 2408 |
| 12 | (sf8 or sf 8 or sf eight or sfeight or shortform 8 or shortform 8 or shortform8 or short form8 or shortform eight or short form eight).ti,ab,kw. | 574 |
| 13 | (sf12 or sf 12 or short form 12 or shortform 12 or short form12 or shortform12 or sf twelve or sftwelve or shortform twelve or short form twelve).ti,ab,kw. | 6861 |
| 14 | (sf16 or sf 16 or short form 16 or shortform 16 or short form16 or shortform16 or sf sixteen or sfsixteen or shortform sixteen or short form sixteen).ti,ab,kw. | 37 |
| 15 | (sf20 or sf 20 or short form 20 or shortform 20 or short form20 or shortform20 or sf twenty or sftwenty or shortform twenty or short form twenty).ti,ab,kw. | 433 |
| 16 | (hql or hqol or h qol or hrqol or hr qol).ti,ab,kw. | 21007 |
| 17 | (pqol or qls).ti,ab,kw. | 424 |
| 18 | (quality of wellbeing or quality of well being or index of wellbeing or index of well being or qwb).ti,ab,kw. | 651 |
| 19 | nottingham health profile*.ti,ab,kw. | 1204 |
| 20 | sickness impact profile.ti,ab,kw. | 1080 |
| 21 | Sickness Impact Profile/ | 7292 |
| 22 | (eq or euroqol or euro qol or eq5d or eq 5d or euroqual or euro qual).ti,ab,kw. | 19504 |
| 23 | duke health profile.ti,ab,kw. | 90 |
| 24 | functional status questionnaire.ti,ab,kw. | 128 |
| 25 | dartmouth coop functional health assessment*.ti,ab,kw. | 13 |
| 26 | (PROMIS or "Patient-Reported Outcomes Measurement Information System").ti,ab. | 3118 |
| 27 | Visual Analog Scale/ | 4015 |
| 28 | (visual analogue scale or visual analog scale).ti,ab,kw. | 58924 |
| 29 | exp Patient Reported Outcome Measures/ | 11196 |
| 30 | ("patient reported" or "patient-reported").ti,ab. | 43338 |
| 31 | (humanistic adj3 (burden or impact or outcome)).ti,ab. | 307 |
| 32 | ((Disability and Impact Profile) or Disability Impact Profile).ti,ab,kw. | 522 |
| 33 | ("Performance Scale*" or "treatment satisfaction" or "Computerized Adaptive Testing" or "Empowerment Scale" or "impact survey").ti,ab. | 7008 |
| 34 | (Beck Depression Inventory or Appraisal of Diabetes Scale or Diabetes 39 or Audit of Diabetes-Dependent Quality of Life or ADDQoL or Diabetes Quality of Life Measure or DQoL or eDiabetes Health Profile or Diabetes Quality of Life Clinical Trial Questionnaire or Barriers to Physical Activity in Diabetes or Diabetes Obstacles Questionnaire or Diabetes Treatment Satisfaction Questionnaire or DTSQ or Diabetes Treatment Satisfaction Questionnaire for Inpatients or Diabetes Symptom Checklist-Revised or Diabetes Computerized Adaptive Testing or Diabetes Impact Survey or Insulin Treatment Satisfaction Questionnaire or Diabetes Empowerment Scale or Diabetes Specific Quality of Life Questionnaire or Satisfaction with Oral Anti-Diabetic Agent Scale).ti,ab. | 14398 |
| 35 | or/6-34 | 543307 |
| 36 | 5 and 35 | 3596 |
| 37 | exp Animals/ not exp Humans/ | 4976850 |
| 38 | (book or chapter or editorial or erratum or note or short survey or tombstone or comment).pt. | 1367851 |
| 39 | Case Reports/ | 2256717 |
| 40 | case report.tw. | 357800 |
| 41 | or/37-40 | 8564827 |
| 42 | 36 not 41 | 3510 |
| 43 | ("paediatric*" or "pediatric*" or "child*" or "kid*" or "young adult*" or "teen*" or "adolescen*" or "boy*" or "girl*" or "youngster*" or "juvenil*" or "minor*" or "boyhood" or "schoolchild*" or "school child*" or "youth*" or "under*age" or "pubescen*" or "school*" or "prematur*" or "preterm*").ti,ab. | 3237335 |
| 44 | exp Child/ | 2059393 |
| 45 | 43 or 44 | 4054562 |
| 46 | 42 and 45 | 1603 |

**Table A2.3**: Search strategy for PsycInfo via EBSCOhost

| **Database: PsycInfo**  **Search executed: March 17, 2022** | | | |
| --- | --- | --- | --- |
| **#** | **String** | **Limiter/Expanders** | **Hits** |
| 1 | ( ( type 1 diabetes OR type I diabetes OR insulin-dependent OR juvenile diabetes OR T1DM ORT1D) NOT animal ) AND (quality of life OR (patient reported OR patient-reported) OR (sf36 or sf 36 or short form 36 or shortform 36 or short form36 or shortform36 or sf thirtysix or sfthirtysix or sfthirty six or sf thirty six or shortform thirtysix or shortform thirty six or short form thirtysix or short form thirty six) OR (sf6 or sf 6 or short form 6 or shortform 6 or sf six or sfsix or shortform six or short form six or shortform6 or short form6) OR (sf8 or sf 8 or sf eight or sfeight or shortform 8 or shortform 8 or shortform8 or short form8 or shortform eight or short form eight) OR (sf12 or sf 12 or short form 12 or shortform 12 or short form12 or shortform12 or sf twelve or sftwelve or shortform twelve or short form twelve) OR (sf16 or sf 16 or short form 16 or shortform 16 or short form16 or shortform16 or sf sixteen or sfsixteen or shortform sixteen or short form sixteen) OR (sf20 or sf 20 or short form 20 or shortform 20 or short form20 or shortform20 or sf twenty or sftwenty or shortform twenty or short form twenty) OR (hql or hqol or h qol or hrqol or hr qol) OR (pqol or qls) OR (quality of wellbeing or quality of well being or index of wellbeing or index of well being or qwb) OR nottingham health profile OR sickness impact profile OR (eq or euroqol or euro qol or eq5d or eq 5d or euroqual or euro qual) OR duke health profile OR functional status questionnaire OR dartmouth coop functional health assessment OR (PROMIS or "Patient-Reported Outcomes Measurement Information System") OR (visual analogue scale or visual analog scale) OR (humanistic burden or humanistic impact or humanistic outcome)) OR ((Disability and Impact Profile) or Disability Impact Profile) OR ("Performance Scale" or “treatment satisfaction" or “Computerized Adaptive Testing" or "Empowerment Scale" or "impact survey") OR (Beck Depression Inventory or Appraisal of Diabetes Scale or Diabetes 39 or Audit of Diabetes-Dependent Quality of Life or ADDQoL or Diabetes Quality of Life Measure or DQoL or eDiabetes Health Profile or Diabetes Quality of Life Clinical Trial Questionnaire or Barriers to Physical Activity in Diabetes or Diabetes Obstacles Questionnaire or Diabetes Treatment Satisfaction Questionnaire or DTSQ or Diabetes Treatment Satisfaction Questionnaire for Inpatients or Diabetes Symptom Checklist-Revised or Diabetes Computerized Adaptive Testing or Diabetes Impact Survey or Insulin Treatment Satisfaction Questionnaire or Diabetes Empowerment Scale or Diabetes Specific Quality of Life Questionnaire or Satisfaction with Oral Anti-Diabetic Agent Scale) ) | Limiters - Publication Type: All Journals; English; Language: English; Age Groups: School Age (6-12 yrs), Adolescence (13-17 yrs), Young Adulthood (18-29yrs); Population Group: Human  Expanders - Apply equivalent subjects  Search modes  - Boolean/Phrase | 692 |

**Table A2.4**: Search strategy for conference proceedings

| **Conference proceedings**  **Search executed: April 20, 2022** | | | |
| --- | --- | --- | --- |
| **Conference** | **Search String** | **Hits** | **Included** |
| American Diabetes Association (2020 – 2022) | type 1 diabetes AND pediatric AND newly diagnosed | 224 | 0 |
|  | T1D AND quality of life | 105 | 0 |
| European Association for the Study of Diabetes (2020 – 2022) | type 1 OR T1D AND quality of life | 25 | 0 |
|  | type 1 OR T1D AND pediatric | 10 | 0 |
| International Society for Pediatric and Adolescent Diabetes (2020 – 2022) | N/A* | N/A | 0 |

*All abstracts were reviewed. N/A, not applicable.

**Search strategies for patients diagnosed with T1D of any duration SLR**

**Table A2.5**: Search strategy for Embase via OvidSP

| **Database: Embase 1974 to 2022 March 22** | | |
| --- | --- | --- |
| **Search executed: March 23, 2022** | | |
| **#** | **String** | **Hits** |
| 1 | exp insulin dependent diabetes mellitus/ | 127729 |
| 2 | (((Type* adj3 "1") or "I" or one*) adj3 (diabete* or diabetic*)).ti,ab. | 111374 |
| 3 | (insulin* adj3 depend* adj3 (diabete* or diabetic*)).ti,ab. | 31561 |
| 4 | (type 1 diabete$ or type 1 diabetic* or diabetes mellitus, type 1 or juvenile diabete$ or insulin dependent diabete$ mellitus).ti,ab. | 95785 |
| 5 | or/1-4 | 178953 |
| 6 | socioeconomics/ | 151415 |
| 7 | exp Quality of Life/ | 572480 |
| 8 | quality of life.ti,kw. | 155198 |
| 9 | ((instrument or instruments) adj3 quality of life).ab. | 5088 |
| 10 | (sf36 or sf 36 or short form 36 or shortform 36 or short form36 or shortform36 or sf thirtysix or sfthirtysix or sfthirty six or sf thirty six or shortform thirtysix or shortform thirty six or short form thirtysix or short form thirty six).ti,ab,kw. | 46285 |
| 11 | (sf6 or sf 6 or short form 6 or shortform 6 or sf six or sfsix or shortform six or short form six or shortform6 or short form6).ti,ab,kw. | 2700 |
| 12 | (sf8 or sf 8 or sf eight or sfeight or shortform 8 or shortform 8 or shortform8 or short form8 or shortform eight or short form eight).ti,ab,kw. | 949 |
| 13 | (sf12 or sf 12 or short form 12 or shortform 12 or short form12 or shortform12 or sf twelve or sftwelve or shortform twelve or short form twelve).ti,ab,kw. | 10982 |
| 14 | (sf16 or sf 16 or short form 16 or shortform 16 or short form16 or shortform16 or sf sixteen or sfsixteen or shortform sixteen or short form sixteen).ti,ab,kw. | 64 |
| 15 | (sf20 or sf 20 or short form 20 or shortform 20 or short form20 or shortform20 or sf twenty or sftwenty or shortform twenty or short form twenty).ti,ab,kw. | 489 |
| 16 | (hql or hqol or h qol or hrqol or hr qol).ti,ab,kw. | 34134 |
| 17 | (pqol or qls).ti,ab,kw. | 694 |
| 18 | (quality of wellbeing or quality of well being or index of wellbeing or index of well being or qwb).ti,ab,kw. | 811 |
| 19 | nottingham health profile*.ti,ab,kw. | 1615 |
| 20 | nottingham health profile/ | 580 |
| 21 | sickness impact profile.ti,ab,kw. | 1268 |
| 22 | sickness impact profile/ | 2361 |
| 23 | (eq or euroqol or euro qol or eq5d or eq 5d or euroqual or euro qual).ti,ab,kw. | 32735 |
| 24 | duke health profile.ti,ab,kw. | 115 |
| 25 | functional status questionnaire.ti,ab,kw. | 165 |
| 26 | dartmouth coop functional health assessment*.ti,ab,kw. | 13 |
| 27 | (PROMIS or "Patient-Reported Outcomes Measurement Information System").ti,ab. | 6245 |
| 28 | Visual Analog Scale/ | 104864 |
| 29 | (visual analogue scale or visual analog scale).ti,ab,kw. | 82693 |
| 30 | exp patient-reported outcome/ | 38891 |
| 31 | ("patient reported" or "patient-reported").ti,ab. | 78104 |
| 32 | (humanistic adj3 (burden or impact or outcome)).ti,ab. | 874 |
| 33 | ((Disability and Impact Profile) or Disability Impact Profile).ti,ab,kw. | 601 |
| 34 | ("Performance Scale*" or "treatment satisfaction" or "Computerized Adaptive Testing" or "Empowerment Scale" or "impact survey").ti,ab. | 11126 |
| 35 | (Beck Depression Inventory or Appraisal of Diabetes Scale or Diabetes 39 or Audit of Diabetes-Dependent Quality of Life or ADDQoL or Diabetes Quality of Life Measure or DQoL or eDiabetes Health Profile or Diabetes Quality of Life Clinical Trial Questionnaire or Barriers to Physical Activity in Diabetes or Diabetes Obstacles Questionnaire or Diabetes Treatment Satisfaction Questionnaire or DTSQ or Diabetes Treatment Satisfaction Questionnaire for Inpatients or Diabetes Symptom Checklist-Revised or Diabetes Computerized Adaptive Testing or Diabetes Impact Survey or Insulin Treatment Satisfaction Questionnaire or Diabetes Empowerment Scale or Diabetes Specific Quality of Life Questionnaire or Satisfaction with Oral Anti-Diabetic Agent Scale).ti,ab. | 22295 |
| 36 | or/6-35 | 946347 |
| 37 | 5 and 36 | 8045 |
| 38 | (exp animal/ or nonhuman/) not exp human/ | 6770592 |
| 39 | (book or chapter or editorial or erratum or letter or note or short survey or tombstone or comment).pt. | 3487948 |
| 40 | Case Study/ | 84432 |
| 41 | case report.tw. | 480140 |
| 42 | or/38-41 | 10624594 |
| 43 | (conference or conference abstract or conference review).pt. | 5127456 |
| 44 | limit 43 to yr="2020 -Current" | 553563 |
| 45 | 43 not 44 | 4573893 |
| 46 | 42 or 45 | 14546728 |
| 47 | 37 not 46 | 5445 |
| 48 | ("paediatric*" or "pediatric*" or "child*" or "kid*" or "young adult*" or "teen*" or "adolescen*" or "boy*" or "girl*" or "youngster*" or "juvenil*" or "minor*" or "boyhood" or "schoolchild*" or "school child*" or "youth*" or "under*age" or "pubescen*" or "school*" or "prematur*" or "preterm*").ti,ab. | 4132024 |
| 49 | exp child/ | 2858586 |
| 50 | 48 or 49 | 5186129 |
| 51 | 47 and 50 | 2101 |

**Table A2.6**: Search strategy for MEDLINE® via OvidSP

| **Database: MEDLINE® 1946 to March 22, 2022** | | |
| --- | --- | --- |
| **Search executed: March 23, 2022** | | |
| **#** | **String** | **Hits** |
| 1 | exp Diabetes Mellitus, Type 1/ | 82163 |
| 2 | (((Type* adj3 "1") or "I" or one*) adj3 (diabete* or diabetic*)).ti,ab. | 71497 |
| 3 | (insulin* adj3 depend* adj3 (diabete* or diabetic*)).ti,ab. | 26770 |
| 4 | (type 1 diabete$ or type 1 diabetic* or diabetes mellitus, type 1 or juvenile diabete$ or insulin dependent diabete$ mellitus).ti,ab. | 65237 |
| 5 | or/1-4 | 123895 |
| 6 | Socioeconomic Factors/ | 168487 |
| 7 | exp "Quality of Life"/ | 235979 |
| 8 | quality of life.ti,kw. | 98434 |
| 9 | ((instrument or instruments) adj3 quality of life).ab. | 3698 |
| 10 | (sf36 or sf 36 or short form 36 or shortform 36 or short form36 or shortform36 or sf thirtysix or sfthirtysix or sfthirty six or sf thirty six or shortform thirtysix or shortform thirty six or short form thirtysix or short form thirty six).ti,ab,kw. | 28517 |
| 11 | (sf6 or sf 6 or short form 6 or shortform 6 or sf six or sfsix or shortform six or short form six or shortform6 or short form6).ti,ab,kw. | 2408 |
| 12 | (sf8 or sf 8 or sf eight or sfeight or shortform 8 or shortform 8 or shortform8 or short form8 or shortform eight or short form eight).ti,ab,kw. | 574 |
| 13 | (sf12 or sf 12 or short form 12 or shortform 12 or short form12 or shortform12 or sf twelve or sftwelve or shortform twelve or short form twelve).ti,ab,kw. | 6861 |
| 14 | (sf16 or sf 16 or short form 16 or shortform 16 or short form16 or shortform16 or sf sixteen or sfsixteen or shortform sixteen or short form sixteen).ti,ab,kw. | 37 |
| 15 | (sf20 or sf 20 or short form 20 or shortform 20 or short form20 or shortform20 or sf twenty or sftwenty or shortform twenty or short form twenty).ti,ab,kw. | 433 |
| 16 | (hql or hqol or h qol or hrqol or hr qol).ti,ab,kw. | 21007 |
| 17 | (pqol or qls).ti,ab,kw. | 424 |
| 18 | (quality of wellbeing or quality of well being or index of wellbeing or index of well being or qwb).ti,ab,kw. | 651 |
| 19 | nottingham health profile*.ti,ab,kw. | 1204 |
| 20 | sickness impact profile.ti,ab,kw. | 1080 |
| 21 | Sickness Impact Profile/ | 7292 |
| 22 | (eq or euroqol or euro qol or eq5d or eq 5d or euroqual or euro qual).ti,ab,kw. | 19504 |
| 23 | duke health profile.ti,ab,kw. | 90 |
| 24 | functional status questionnaire.ti,ab,kw. | 128 |
| 25 | dartmouth coop functional health assessment*.ti,ab,kw. | 13 |
| 26 | (PROMIS or "Patient-Reported Outcomes Measurement Information System").ti,ab. | 3118 |
| 27 | Visual Analog Scale/ | 4015 |
| 28 | (visual analogue scale or visual analog scale).ti,ab,kw. | 58924 |
| 29 | exp Patient Reported Outcome Measures/ | 11196 |
| 30 | ("patient reported" or "patient-reported").ti,ab. | 43338 |
| 31 | (humanistic adj3 (burden or impact or outcome)).ti,ab. | 307 |
| 32 | ((Disability and Impact Profile) or Disability Impact Profile).ti,ab,kw. | 522 |
| 33 | ("Performance Scale*" or "treatment satisfaction" or "Computerized Adaptive Testing" or "Empowerment Scale" or "impact survey").ti,ab. | 7008 |
| 34 | (Beck Depression Inventory or Appraisal of Diabetes Scale or Diabetes 39 or Audit of Diabetes-Dependent Quality of Life or ADDQoL or Diabetes Quality of Life Measure or DQoL or eDiabetes Health Profile or Diabetes Quality of Life Clinical Trial Questionnaire or Barriers to Physical Activity in Diabetes or Diabetes Obstacles Questionnaire or Diabetes Treatment Satisfaction Questionnaire or DTSQ or Diabetes Treatment Satisfaction Questionnaire for Inpatients or Diabetes Symptom Checklist-Revised or Diabetes Computerized Adaptive Testing or Diabetes Impact Survey or Insulin Treatment Satisfaction Questionnaire or Diabetes Empowerment Scale or Diabetes Specific Quality of Life Questionnaire or Satisfaction with Oral Anti-Diabetic Agent Scale).ti,ab. | 14398 |
| 35 | or/6-34 | 543307 |
| 36 | 5 and 35 | 3596 |
| 37 | exp Animals/ not exp Humans/ | 4976850 |
| 38 | (book or chapter or editorial or erratum or letter or note or short survey or tombstone or comment).pt. | 2046109 |
| 39 | Case Reports/ | 2256717 |
| 40 | case report.tw. | 357800 |
| 41 | or/37-40 | 9001906 |
| 42 | 36 not 41 | 3463 |
| 43 | ("paediatric*" or "pediatric*" or "child*" or "kid*" or "young adult*" or "teen*" or "adolescen*" or "boy*" or "girl*" or "youngster*" or "juvenil*" or "minor*" or "boyhood" or "schoolchild*" or "school child*" or "youth*" or "under*age" or "pubescen*" or "school*" or "prematur*" or "preterm*").ti,ab. | 3237335 |
| 44 | exp Child/ | 2059393 |
| 45 | 43 or 44 | 4054562 |
| 46 | 42 and 45 | 1596 |

**Table A2.7**: Search strategy for PsycInfo via EBSCOHost

| **Database: PsycInfo** | | |
| --- | --- | --- |
| **Search executed: March 23, 2022** | | |
| **#** | **String** | **Hits** |
| 1 | ( ( type 1 diabetes OR type I diabetes OR insulin-dependent OR juvenile diabetes OR T1DM ORT1D) NOT animal ) AND (quality of life OR (patient reported OR patient-reported) OR (sf36 or sf36 or short form 36 or shortform 36 or shortform36 or shortform36 or sf thirtysix or sfthirtysix or sfthirty six or sf thirty six or shortform thirtysix or shortform thirty six orshort form thirtysix or short form thirty six) OR (sf6 or sf 6 or short form 6 or shortform 6 or sf six or sfsix or shortform six or short form six orshortform6 or short form6) OR (sf8 or sf 8 or sf eight or sfeight or shortform 8 or shortform 8 or shortform8 or short form8 or shortform eight or shortform eight) OR (sf12 or sf12 or short form 12 or shortform 12 or shortform12 or shortform12 or sf twelve or sftwelve orshortform twelve or shortform twelve) OR (sf16 orsf 16 or short form 16 or shortform 16 or shortform16 or shortform16 or sf sixteen or sfsixteen or shortform sixteen or shortform sixteen) OR (sf20 or sf 20 or short form 20 or shortform 20 or shortform20 or shortform20 or sf twenty or sftwenty or shortform twenty or shortform twenty) OR (hql or hqol or h qol or hrqol or hrqol) OR (pqol or qls) OR (quality of wellbeing or quality of well being or index of wellbeing or index of well being or qwb) OR nottingham health profile OR sickness impactprofile OR (eq or euroqol or euro qol or eq5d or eq5d or euroqual or euroqual) OR duke health profile OR functional status questionnaire OR dartmouth coop functional health assessment OR(PROMIS or "Patient-Reported Outcomes Measurement Information System") OR (visual analogue scale or visual analog scale) OR (humanistic burden or humanistic impact or humanistic outcome)) OR ((Disability and Impact Profile) or Disability Impact Profile) OR ("Performance Scale" or "treatment satisfaction" or "Computerized Adaptive Testing" or "Empowerment Scale" or "impact survey") OR (Beck Depression Inventory or Appraisal of Diabetes Scale or Diabetes 39 or Audit of Diabetes-Dependent Quality of Life or ADDQoL or Diabetes Quality of Life Measure or DQoL or eDiabetes Health Profile or Diabetes Quality of Life Clinical Trial Questionnaire or Barriers to Physical Activity in Diabetes or Diabetes Obstacles Questionnaire or Diabetes Treatment Satisfaction Questionnaire or DTSQ or Diabetes Treatment Satisfaction Questionnaire for Inpatients or Diabetes Symptom Checklist-Revised or Diabetes Computerized Adaptive Testing or Diabetes Impact Survey or Insulin Treatment Satisfaction Questionnaire or Diabetes Empowerment Scale or Diabetes Specific Quality of Life Questionnaire or Satisfaction with Oral Anti-Diabetic Agent Scale) ) | 692 |

**Table A2.8**: Search strategy for grey literature search

| **No.** | **Website** | **Terms** | **Hits** | **Relevant** |
| --- | --- | --- | --- | --- |
| 1 | American Diabetes Association - https://professional.diabetes.org/search/site/T1D%2520and%2520quality%2520of%2520life | type 1 diabetes and pediatric | 224 | 0 |
| 2 | American Diabetes Association - https://www.diabetes.org/ | T1D and quality of life | 105 | 0 |
| 3 | European Association for the Study of Diabetes - https://www.easd.org/virtualmeeting/home.html#!resourcegroups/order=playbacks_count&event_ids=16&page=1&group=&resourcetype_ids=&tag_ids=&query=type%201%20or%20T1D%20and%20quality%20of%20life | type 1 or T1D and quality of life | 25 | 0 |
| 4 | European Association for the Study of Diabetes - https://www.easd.org/virtualmeeting/home.html#!resourcegroups/order=playbacks_count&event_ids=16&page=1&group=&resourcetype_ids=&tag_ids=&query=type%201%20or%20T1D%20and%20quality%20of%20life | type 1 or T1D and pediatric | 10 | 0 |
| 5 | International Society for Pediatric and Adolescent Diabetes (ISPAD) - https://www.ispad.org/page/annualmeetings | N/A* | N/A* | 0 |

*A review of all abstracts from annual meetings (2020, 2021) was conducted. N/A, not applicable.

**Search strategies for caregivers of patients newly diagnosed with T1D SLR**

**Table A2.9**: Search strategy for MEDLINE®

| **Database: MEDLINE®** | | |
| --- | --- | --- |
| **Search executed: December 8, 2021** | | |
| **#** | **String** | **Hits** |
| 1 | exp insulin dependent diabetes mellitus/ | 80740 |
| 2 | (((Type* adj3 "1") or "I" or one*) adj3 (diabete* or diabetic*)).ti,ab. | 70248 |
| 3 | (insulin* adj3 depend* adj3 (diabete* or dibetic*)).ti,ab. | 21724 |
| 4 | (type 1 diabete$ or type 1 diabetic* or diabetes mellitus, type 1 or juvenile diabete$ or insulin dependent diabete$ mellitus).ti,ab. | 64254 |
| 5 | or/1-4 | 120112 |
| 6 | exp caregiver/ | 43478 |
| 7 | ("caregiver*" or "care partner*" or "care giver*" or "caretaker*" or "care taker*" or "care giving" or "caregiving" or "take care" or "taking care" or "caring for").ti,ab. | 134465 |
| 8 | (("parent*" or "guardian*" or "mother*" or "father*" or "spouse*" or "husband*" or "wife*" or "partner*" or "family member*") adj3 ("car*" or "burden*")).ti,ab. | 41931 |
| 9 | or/6-8 | 171766 |
| 10 | 5 and 9 | 1067 |
| 11 | limit 10 to english language | 1022 |

**Table A2.10**: Search strategy for Embase

| **Database: Embase** | | |
| --- | --- | --- |
| **Search executed: December 8, 2021** | | |
| **#** | **String** | **Hits** |
| 1 | exp insulin dependent diabetes mellitus/ | 125511 |
| 2 | (((Type* adj3 "1") or "I" or one*) adj3 (diabete* or diabetic*)).ti,ab. | 109412 |
| 3 | (insulin* adj3 depend* adj3 (diabete* or dibetic*)).ti,ab. | 25504 |
| 4 | (type 1 diabete$ or type 1 diabetic* or diabetes mellitus, type 1 or juvenile diabete$ or insulin dependent diabete$ mellitus).ti,ab. | 94277 |
| 5 | or/1-4 | 172963 |
| 6 | exp caregiver/ | 94461 |
| 7 | ("caregiver*" or "care partner*" or "care giver*" or "caretaker*" or "care taker*" or "care giving" or "caregiving" or "take care" or "taking care" or "caring for").ti,ab. | 181808 |
| 8 | (("parent*" or "guardian*" or "mother*" or "father*" or "spouse*" or "husband*" or "wife*" or "partner*" or "family member*") adj3 ("car*" or "burden*")).ti,ab. | 55253 |
| 9 | or/6-8 | 236086 |
| 10 | 5 and 9 | 1864 |
| 11 | limit 10 to english language | 1787 |

**Table A2.11**: Search strategy for PsycInfo

| **Database: PsycInfo** | | | |
| --- | --- | --- | --- |
| **Search executed: December 6, 2021** | | | |
| **#** | **Query** | **Limiters/Expanders** | **Hits** |
| S1 | ((type 1 OR type I OR type one) AND (diabete*OR diabetic*)) OR insulin dependent diabetes mellitus OR juvenile diabetes) | Expanders - Apply equivalent subjects  Search modes - Boolean/Phrase | 4790 |
| S2 | MM "Caregivers" | Expanders - Apply equivalent subjects  Search modes - Boolean/Phrase | 24103 |
| S3 | TI ( "caregiver*" or "care partner*" or "care giver*"or "caretaker*" or "caretaker*" or "caregiving" or "caregiving" or "take care" or "taking care" or "caring for") ORAB ( "caregiver*" or "care partner*" or "care giver*"or "caretaker*" or "caretaker*" or "caregiving" or "caregiving" or "take care" or "taking care" or "caring for") | Expanders - Apply equivalent subjects  Search modes - Boolean/Phrase | 88429 |
| S4 | TI ( "parent*" or "guardian*" or "mother*"or "father*" or "spouse*" or "husband*" or "wife*" or "partner*" or "family member*") OR AB ("parent*" or "guardian*" or "mother*" or "father*" or "spouse*" or "husband*"or "wife*" or "partner*" or "family member*") | Expanders - Apply equivalent subjects  Search modes - Boolean/Phrase | 523352 |
| S5 | S2 OR S3 OR S4 | Expanders - Apply equivalent subjects  Search modes - Boolean/Phrase | 579281 |
| S6 | S1 AND S5 | Expanders - Apply equivalent subjects  Search modes - Boolean/Phrase | 1313 |
| S7 | S1 AND S5 | Expanders - Apply equivalent subjects  Narrow by Language: - english  Search modes - Boolean/Phrase | 1273 |

**Search strategies for caregivers of patients with T1D of any duration SLR**

**Table A2.12**: Search strategy for Embase via OvidSP

| **Database: Embase 1974 to December 7, 2021**  **Search executed: December 8, 2021** | | |
| --- | --- | --- |
| **#** | **String** | **Hits** |
| 1 | exp insulin dependent diabetes mellitus/ | 125511 |
| 2 | (((Type* adj3 "1") or "I" or one*) adj3 (diabete* or diabetic*)).ti,ab. | 109412 |
| 3 | (insulin* adj3 depend* adj3 (diabete* or dibetic*)).ti,ab. | 25504 |
| 4 | (type 1 diabete$ or type 1 diabetic* or diabetes mellitus, type 1 or juvenile diabete$ or insulin dependent diabete$ mellitus).ti,ab. | 94277 |
| 5 | or/1-4 | 172963 |
| 6 | exp caregiver/ | 94461 |
| 7 | ("caregiver*" or "care partner*" or "care giver*" or "caretaker*" or "care taker*" or "care giving" or "caregiving" or "take care" or "taking care" or "caring for").ti,ab. | 181808 |
| 8 | (("parent*" or "guardian*" or "mother*" or "father*" or "spouse*" or "husband*" or "wife*" or "partner*" or "family member*") adj3 ("car*" or "burden*")).ti,ab. | 55253 |
| 9 | or/6-8 | 236086 |
| 10 | 5 and 9 | 1864 |
| 11 | limit 10 to english language | 1787 |

**Table A2.13**: Search strategy for MEDLINE® via OvidSP

| **Database: MEDLINE® 1946 to December 7, 2021**  **Search executed: December 8, 2021** | | |
| --- | --- | --- |
| **#** | **String** | **Hits** |
| 1 | exp insulin dependent diabetes mellitus/ | 80740 |
| 2 | (((Type* adj3 "1") or "I" or one*) adj3 (diabete* or diabetic*)).ti,ab. | 70248 |
| 3 | (insulin* adj3 depend* adj3 (diabete* or dibetic*)).ti,ab. | 21724 |
| 4 | (type 1 diabete$ or type 1 diabetic* or diabetes mellitus, type 1 or juvenile diabete$ or insulin dependent diabete$ mellitus).ti,ab. | 64254 |
| 5 | or/1-4 | 120112 |
| 6 | exp caregiver/ | 43478 |
| 7 | ("caregiver*" or "care partner*" or "care giver*" or "caretaker*" or "care taker*" or "care giving" or "caregiving" or "take care" or "taking care" or "caring for").ti,ab. | 134465 |
| 8 | (("parent*" or "guardian*" or "mother*" or "father*" or "spouse*" or "husband*" or "wife*" or "partner*" or "family member*") adj3 ("car*" or "burden*")).ti,ab. | 41931 |
| 9 | or/6-8 | 171766 |
| 10 | 5 and 9 | 1067 |
| 11 | limit 10 to english language | 1022 |

**Table A2.14**: Search strategy for PsycInfo via EBSCOhost

| **Database: PsycInfo**  **Search executed: December 6, 2021** | | |
| --- | --- | --- |
| **#** | **String** | **Hits** |
| S1 | ((type 1 OR type I OR type one) AND (diabete* OR diabetic*)) OR insulin dependent diabetes mellitus OR juvenile diabetes) | 4,790 |
| S2 | MM "Caregivers" | 24,103 |
| S3 | TI ("caregiver*" or "care partner*" or "care giver*" or "caretaker*" or "caretaker*" or "care giving" or "caregiving" or "take care" or "taking care" or "caring for") OR AB ("caregiver*" or "care partner*" or "care giver*" or "caretaker*" or "caretaker*" or "care giving" or "caregiving" or "take care" or "taking care" or "caring for") | 88,429 |
| S4 | TI ("parent*" or "guardian*" or "mother*" or "father*" or "spouse*" or "husband*" or "wife*" or "partner*" or "family member*") OR AB ("parent*" or "guardian*" or "mother*" or "father*" or "spouse*" or "husband*" or "wife*" or "partner*" or "family member*") | 523,352 |
| S5 | S2 OR S3 OR S4 | 579,281 |
| S6 | S1 AND S5 | 1,313 |
| S7 | Limit S6 to English | 1,273 |

**Table A2.15**: Search strategy for gray literature

| **Source** | **Search terms** | **Hits** |
| --- | --- | --- |
| ADA 2019 | Caregiver | 9 |
|  | Partner | 4 |
|  | Parent | 13 |
| EASD 2019 | Type 1 diabetes caregiver | 4 |
| EASD 2020 | Type 1 diabetes caregiver | 4 |
| EASD 2021 | Type 1 diabetes caregiver | 1 |

ADA: American Diabetes Association; EASD: European Association for the Study of Diabetes
