# Supplementary material for: Humanistic burden of pediatric type 1 diabetes on children and informal caregivers: systematic literature reviews
Source: Diabetol Metab Syndr. 2024 Mar 21;16:73. doi: 10.1186/s13098-024-01310-2 (PMC10956250; doi:10.1186/s13098-024-01310-2)
Supplement: Supplementary file 3 — Additional File 3: Study selection (PRISMA flow diagrams and lists of included studies from the systematic literature reviews) [file 13098_2024_1310_MOESM3_ESM.docx]

# Additional File 3: Study selection

**Figure A3.1**: PRISMA flow diagram for patients newly diagnosed with T1D SLR

**Identification of studies via databases and registers**

**Identification**

Records identified from:

Embase (n = 2,124)

MEDLINE® (n = 1,603)

PsycInfo (n = 692)

Records removed *before screening*:

Duplicate records removed (n = 1,111)

Records excluded:

Population (n = 1,408)

Other (n = 723)

Study Design (n = 988)

Duplicate Publication (n = 25)

Included for Library (n = 15)

Records screened

(n = 3,308)

Reports sought for retrieval

(n = 149)

**Screening**

Reports assessed for eligibility

(n =149)

Reports excluded:

Population (n = 130)

Outcomes (n = 12)

Study design (n = 2)

Reports of included studies (n = 5)

**Included**

**Table A3.1**: List of included studies for patients newly diagnosed with T1D SLR

| **First Author & Year** | **Title** |
| --- | --- |
| Clapin 2017 | Home-based vs inpatient education for children newly diagnosed with type 1 diabetes |
| Hiers 2020 | Introducing continuous glucose monitoring technology at diagnosis in pediatric type 1 diabetes: A proof-of-concept study |
| Northam 1996 | Psychosocial and family functioning in children with insulin-dependent diabetes at diagnosis and one year later |
| Kovacs 1986 | Children's self-reports of psychologic adjustment and coping strategies during first year of insulin-dependent diabetes mellitus |
| Yi-Frazier 2018 | Trajectories of acute diabetes-specific stress in adolescents with type 1 diabetes and their caregivers within the first year of diagnosis |

**Figure A3.2**: PRISMA flow diagram for patients diagnosed with T1D of any duration SLR

**Identification of studies via databases and registers**

**Identification**

Records identified from:

Embase (n = 2,124)

MEDLINE® (n = 1,603)

PsycInfo (n = 692)

Records removed *before screening*:

Duplicate records removed (n = 1,111)

Records excluded:

Study design (n = 1,620)

Population (n = 1,019)

Other (n = 452)​

Duplicate publication (n = 20)

Included for summary of intervention characteristics (n = 9)

Included for library (n = 1)

Records screened

(n = 3,308)

Reports sought for retrieval

(n = 187)

**Screening**

Reports excluded:

Population (n = 59)

Outcomes (n = 12)

Other (n = 7)

Study design (n = 3)

Duplicate publication (n = 1)

Reports assessed for eligibility

(n = 187)

Reports of included studies

(n = 105) of which 103 unique studies were included in review

**Included**

**Table A3.2**: List of included studies for patients diagnosed with T1D of any duration SLR

| **First Author & Year** | **Title** |
| --- | --- |
| Abualula 2018 | Self-rated health among American adolescents with type 1 diabetes in the t1d exchange clinic registry |
| Adal 2015 | Recognizing the psychosocial aspects of type 1 diabetes in adolescents |
| Al-Akour 2010 | Quality of life and associated factors among Jordanian adolescents with type 1 diabetes mellitus |
| Alaqeel 2021 | High rate of depression among Saudi children with type 1 diabetes |
| AlBuhairan 2016 | Health related quality of life and family impact of type 1 diabetes among adolescents in Saudi Arabia |
| Al-Hayek 2014 | Assessment of health-related quality of life among adolescents with type 1 diabetes mellitus in Saudi Arabia |
| Almeida 2018 | Psychiatric disorders in adolescents with type 1 diabetes: A case-control study |
| Askari 2020 | Health-related quality of life of Pakistani adolescents with type 1 diabetes and their parents |
| Atay 2018 | Comparison of hand function between children with type 1 diabetes mellitus and children without type 1 diabetes mellitus |
| Babler 2015 | Moving the journey towards independence: Adolescents transitioning to successful diabetes self-management |
| Babler 2015 | Normalizing: Adolescent experiences living with type 1 diabetes |
| Babler 2016 | Helping adolescents with type 1 diabetes “figure it out” |
| Boo 2016 | Diabetes-specific quality of life of Korean children and adolescents with type 1 diabetes |
| Boogerd 2015 | Assessment of psychosocial problems in children with type 1 diabetes and their families: The added value of using standardized questionnaires in addition to clinical estimations of nurses and paediatricians |
| Buresova 2008 | Health related quality of life of children and adolescents with type 1 diabetes |
| Butwicka 2016 | Psychiatric disorders and health-related quality of life in children with type 1 diabetes mellitus |
| Caferoglu 2016 | Health-related quality of life and metabolic control in children and adolescents with type 1 diabetes mellitus |
| Cao 2020 | Characterizing diabetes distress in adolescents with type 1 diabetes |
| Chao 2014 | Self-management in early adolescence and differences by age at diagnosis and duration of type 1 diabetes |
| Chaplin 2009 | Assessment of childhood diabetes-related quality-of-life in west Sweden |
| Cobuz 2016 | Life quality of the child with diabetes mellitus |
| Commissariat 2020 | Identity and treatment adherence in predominantly ethnic minority teens and young adults with type 1 diabetes |
| Costa 2015 | Quality of life of adolescents with type 1 diabetes |
| de Wit 2007 | Self-report and parent-report of physical and psychosocial well-being in Dutch adolescents with type 1 diabetes in relation to glycemic control |
| Delamater 2013 | Measurement of diabetes stress in older children and adolescents with type 1 diabetes mellitus |
| Duru 2015 | Quality of life and psychological screening in children with type 1 diabetes and their mothers |
| Elissa 2017 | Societal norms and conditions and their influence on daily life in children with type 1 diabetes in the west bank in Palestine |
| Elissa 2020 | Self-perceived health status and sense of coherence in children with type 1 diabetes in the west bank, Palestine |
| Faro 1999 | The effect of diabetes on adolescents’ quality of life |
| Faulkner 2003 | Quality of life for adolescents with type 1 diabetes: Parental and youth perspectives |
| Firat 2020 | The relationship between diabetes-related factors, family functioning and health-related quality of life in Turkish adolescents with type 1 diabetes mellitus |
| Forsander 2017 | Adolescent life with diabetes-gender matters for level of distress. Experiences from the national TODS study |
| Fortenberry 2014 | Longitudinal trajectories of illness perceptions among adolescents with type 1 diabetes |
| Girma 2021 | Health-related quality of life and its associated factors in children and adolescents with type1 diabetes, Addis Ababa, Ethiopia |
| Goncalves 2016 | Eating-disordered behaviour in adolescents with type 1 diabetes |
| Graue 2003 | Measuring self-reported, health-related, quality of life in adolescents with type 1 diabetes using both generic and disease-specific instruments |
| Grey 1998 | Personal and family factors associated with quality of life in adolescents with diabetes |
| Guo 2013 | Diabetes self-management, depressive symptoms, quality of life and metabolic control in youth with type 1 diabetes in China |
| Guo 2015 | Diabetes self-management, depressive symptoms, metabolic control and satisfaction with quality of life over time in Chinese youth with type 1 diabetes |
| Haas 2020 | Treatment satisfaction correlated with glycaemic control and burden of diabetes in Swedish adolescents with type 1 diabetes |
| Hanna 2014 | Diabetes-related quality of life and the demands and burdens of diabetes care among emerging adults with type 1 diabetes in the year after high school graduation |
| Hapunda 2015 | Living with type 1 diabetes is challenging for Zambian adolescents: Qualitative data on stress, coping with stress and quality of care and life |
| Harrington 2021 | Associations of diabetes self-management characteristics, HbA1c, and psychosocial outcomes with depressive symptoms in a contemporary sample of adolescents with type 1 diabetes |
| Hilliard 2011 | Psychological screening in adolescents with type 1 diabetes predicts outcomes one year later |
| Hilliard 2017 | Strengths, risk factors, and resilient outcomes in adolescents with type 1 diabetes: Results from Diabetes Miles Youth-Australia |
| Hoey 2001 | Good metabolic control is associated with better quality of life in 2,101 adolescents with type 1 diabetes |
| Ingerski 2010 | Correlates of glycemic control and quality of life outcomes in adolescents with type 1 diabetes |
| Insabella 2007 | The transition to young adulthood in youth with type 1 diabetes on intensive treatment |
| Jaser 2008 | Mediators of depressive symptoms in children with type 1 diabetes and their mothers |
| Jaser 2017 | Stress and coping predicts adjustment and glycemic control in adolescents with type 1 diabetes |
| Kalyva 2016 | A cross-cultural study on perceived health-related quality of life in children and adolescents with type 1 diabetes mellitus |
| Kamody 2018 | Psychological flexibility among youth with type 1 diabetes: Relating patterns of acceptance, adherence, and stress to adaptation |
| Kane 2020 | Glycemic control and self-rated health among ethnically diverse adolescents with type 1 diabetes |
| Kovacs 1985 | Initial coping responses and psychosocial characteristics of children with insulin-dependent diabetes mellitus |
| Kovacs 1986 | Children’s self-reports of psychologic adjustment and coping strategies during first year of insulin-dependent diabetes mellitus |
| Kristensen 2014 | Symptoms of emotional, behavioral, and social difficulties in the Danish population of children and adolescents with type 1 diabetes — results of a national survey |
| Kyngas 1995 | Diabetes: An adolescent’s perspective |
| La Greca 1995 | Adolescents with diabetes: Gender differences in psychosocial functioning and glycemic control |
| Laffel 2003 | General quality of life in youth with type 1 diabetes: Relationship to patient management and diabetes-specific family conflict |
| Lopez-Bastida 2019 | Health-related quality of life in type 1 diabetes mellitus pediatric patients and their caregivers in Spain: An observational cross-sectional study |
| Lukacs 2016 | Generic and disease-specific quality of life in adolescents with type 1 diabetes: Comparison to age-matched healthy peers |
| Lukacs 2018 | Health-related quality of life of adolescents with type 1 diabetes in the context of resilience |
| Lukács 2014 | Factors influencing the diabetes-specific health-related quality of life in children and adolescents with type 1 diabetes mellitus |
| Ly 2011 | Neurocognitive outcomes in young adults with early-onset type 1 diabetes: A prospective follow-up study |
| Madrigal 2020 | Type 1 diabetes mellitus in pediatric patients and its impact on relationships in the family environment |
| Malik 2009 | Explaining the adjustment of adolescents with type 1 diabetes: Role of diabetes-specific and psychosocial factors |
| Markosyan 2019 | Health-related quality of life in children with type 1 diabetes in Armenia |
| Marques 2021 | Associated factors with the quality of life of adolescents with type 1 diabetes |
| Moreira 2006 | [living with diabetes: The experience as it is told by children] |
| Moussa 2005 | Social and psychological characteristics of Kuwaiti children and adolescents with type 1 diabetes |
| Mozzillo 2017 | Unhealthy lifestyle habits and diabetes-specific health-related quality of life in youths with type 1 diabetes |
| Muhe-Aldeen 2020 | Mood disorder in adolescents with diabetes mellitus in Kirkuk city |
| Mulyasari 2019 | Coping strategies and quality of life of children with type 1 diabetes mellitus: A preliminary study |
| Murillo 2017 | Health-related quality of life (HRQoL) and its associated factors in children with type 1 diabetes mellitus (T1DM) |
| Nakamura 2010 | Title: Health-related and diabetes-related quality of life (QoL) in Japanese children and adolescents with type 1 and type 2 diabetes |
| Nardi 2008 | Quality of life, psychological adjustment and metabolic control in youths with type 1 diabetes: A study with self- and parent-report questionnaires |
| Naughton 2014 | Longitudinal associations between sex, diabetes self-care, and health-related quality of life among youth with type 1 or type 2 diabetes mellitus |
| Northam 1996 | Psychosocial and family functioning in children with insulin-dependent diabetes at diagnosis and one year later |
| Overgaard 2020 | Disruption, worries and autonomy in the everyday lives of adolescents with type 1 diabetes and their family members: A qualitative study of intrafamilial challenges |
| Ozyazicioglu 2017 | A determination of the quality of life of children and adolescents with type 1 diabetes and their parents |
| Palladino 2013 | Emerging adults with type 1 diabetes: A comparison to peers without diabetes |
| Pinar 2003 | Self-efficacy and its interrelation with family environment and metabolic control in Turkish adolescents with type 1 diabetes |
| Pinar 2022 | Living with type 1 diabetes: A qualitative study |
| Rechenberg 2017 | General and diabetes-specific stress in adolescents with type 1 diabetes |
| Rechenberg 2019 | State and trait anxiety and diabetes outcomes in youth with type 1 diabetes |
| Reid 2013 | Relations between quality of life, family factors, adherence, and glycemic control in pediatric patients with type 1 diabetes mellitus |
| Reitblat 2016 | Life with type 1 diabetes: Views of Hispanic adolescents and their clinicians |
| Rostami 2014 | Supporting adolescents with type 1 diabetes mellitus: A qualitative study |
| Saoji 2018 | The relationship of type 1 diabetes self-management barriers to child and parent quality of life: A us cross-sectional study |
| Sendela 2015 | Prevalence of depressive symptoms in school aged children with type 1 diabetes — a questionnaire study |
| Serrabulho 2012 | The health and lifestyles of adolescents with type 1 diabetes in Portugal |
| Sinnamon 2013 | Differentiating disordered affect in children and adolescents with type 1 diabetes |
| Souza 2019 | Health-related quality of life of adolescents with type 1 diabetes mellitus |
| Stahl 2012 | Health-related quality of life among German youths with early-onset and long-duration type 1 diabetes |
| Stahl-Pehe 2022 | Overestimation and underestimation of youths’ health-related quality of life are associated with youth and caregiver positive screens for depression: Results of a population-based study among youths with longstanding type 1 diabetes |
| Stewart 2005 | Depressive symptoms predict hospitalization for adolescents with type 1 diabetes mellitus |
| Striegel-Moore 1992 | Prevalence of eating disorder symptoms in preadolescent and adolescent girls with IDDM |
| Sullivan 1978 | Self-esteem and depression in adolescent diabetic girls |
| Tercyak 2005 | Health attitudes, beliefs, and risk behaviors among adolescents and young adults with type 1 diabetes |
| Vlahou 2021 | Outpatient clinic-wide psychological screening for children and adolescents with type 1 diabetes in Qatar: An initiative for integrative healthcare in the gulf region |
| Wagner 2004 | Age related differences in individual quality of life domains in youth with type 1 diabetes |
| Weinger 2001 | Adolescent views of diabetes-related parent conflict and support: A focus group analysis |
| Yayan 2019 | The relationship between the quality of life and depression levels of young people with type I diabetes |
| Yi-Frazier 2015 | The association of personal resilience with stress, coping, and diabetes outcomes in adolescents with type 1 diabetes: Variable- and person-focused approaches |
| Yi-Frazier 2018 | Trajectories of acute diabetes-specific stress in adolescents with type 1 diabetes and their caregivers within the first year of diagnosis |

**Figure A3.3**: PRISMA flow diagram for caregivers of patients newly diagnosed with T1D SLR

**Identification of studies via other methods**

**Identification of studies via databases and registers**

Records identified from:

Conference proceedings (n = 36)

Records removed *before screening*:

Duplicate records removed (n = 1,052)

Records identified from:

Embase (n = 1,787)

MEDLINE® (n = 1,022)

PsycInfo (n = 1,273)

Records screened

(n = 3,030)

Reports sought for retrieval

(n = 40)

Reports assessed for eligibility

(n = 39)

**Identification**

Records excluded

(n = 2,990)

Reports not retrieved (n = 1)

Reports sought for retrieval

(n = 36)

**Screening**

Reports excluded:

Population

(n = 17)

Outcomes

(n = 16)

Other (n = 2)

Population

(n = 1)

Reports excluded:

Population

(n = 26)

Outcomes (n = 4)

Study design

(n = 3)

Reports assessed for eligibility

(n = 36)

Reports of included studies

(n = 6) of which 5 unique studies were included in review

**Included**

**Table A3.3**: List of included studies for caregivers of patients newly diagnosed with T1D SLR

| **First Author & Year** | **Title** |
| --- | --- |
| Kovacs 1990 | Psychological functioning among mothers of children with insulin-dependent diabetes mellitus: A longitudinal study |
| Landolt 2002 | Posttraumatic stress disorder in parents of children with newly diagnosed type 1 diabetes |
| Landolt 2005 | Prospective study of posttraumatic stress disorder in parents of children with newly diagnosed type 1 diabetes |
| Northam 1996 | Psychosocial and family functioning in children with insulin-dependent diabetes at diagnosis and one year later |
| Sullivan-Bolyai 2015 | PETS-D: Impact on diabetes management outcomes |
| Yi-Frazier 2018 | Trajectories of acute diabetes-specific stress in adolescents with type 1 diabetes and their caregivers within the first year of diagnosis |

**Figure A3.4**: PRISMA flow diagram for caregivers of patients with T1D of any duration SLR

**Identification of studies via other methods**

**Identification of studies via databases and registers**

**Identification of studies via databases and registers**

**Identification**

Records identified from:

Conference proceedings

(n = 18)

Records removed *before screening*:

Duplicate records removed (n = 1,142)

Records identified from:

Embase (n = 1,787)

MEDLINE® (n = 1,022)

PsycInfo n = 1,273)

Records screened

(n = 2,940)

Reports sought for retrieval

(n = 284)

Records excluded

Population (n = 1,112)​

Other (n = 1,088)​

Study design (n = 453)​

Duplicate publications

(n = 3)​

Reports sought for retrieval

(n = 18)

**Screening**

Reports excluded:

Other (n = 16)

Population (n = 1)

Reports assessed for eligibility

(n = 18)

Reports excluded:

Population (n = 83)

Outcomes (n = 53)

Study design (n = 7)

Reports assessed for eligibility

(n = 284)

Reports of included studies (n = 142) of which 135 unique studies were included in review

**Included**

**Table A3.4**: List of included studies for caregivers of patients with T1D of any duration SLR

| **First Author & Year** | **Title** |
| --- | --- |
| Abitbol 2021 | When low blood sugars cause high anxiety: Fear of hypoglycemia among parents of youth with type 1 diabetes mellitus |
| Afifi 2019 | Testing the theory of resilience and relational load (TRRL) in families with type in diabetes |
| Al Ansari 2021 | Self-reported psychological disorders among the mothers of children with autism spectrum disorder, type 1 diabetes mellitus, and typically developed children |
| Aldubayee 2020 | Parental levels of stress managing a child diagnosed with type 1 diabetes in Riyadh: A cross sectional study |
| Anderson 2009 | Dyadic measures of the parent–child relationship during the transition to adolescence and glycemic control in children with type 1 diabetes |
| Askari 2020 | Health-related quality of life of Pakistani adolescents with type 1 diabetes and their parents |
| Auslander 1997 | Mothers' satisfaction with medical care: Perceptions of racism, family stress, and medical outcomes in children with diabetes |
| Barbed Ferrandez 2021 | Impact on the well-being perceived by caregivers of children and adolescents with type 1 diabetes following the use of interstitial glucose measurement systems |
| Barnard 2016 | Impact of type 1 diabetes technology on family members/significant others of people with diabetes |
| Berg 2007 | Mother-child appraised involvement in coping with diabetes stressors and emotional adjustment |
| Beveridge 2006 | Mother and adolescent representations of illness ownership and stressful events surrounding diabetes |
| Bomba 2018 | Experiences in sensor-augmented pump therapy in families with two children with type 1 diabetes: A qualitative study |
| Boogerd 2017 | Sugarsquare, a web-based patient portal for parents of a child with type 1 diabetes: Multicenter randomized controlled feasibility trial |
| Boogerd 2015 | Assessment of psychosocial problems in children with type 1 diabetes and their families: The added value of using standardized questionnaires in addition to clinical estimations of nurses and pediatricians |
| Boogerd 2015 | Parents’ experiences, needs, and preferences in pediatric diabetes care: Suggestions for improvement of care and the possible role of the internet a qualitative study |
| Bowes 2009 | Chronic sorrow in parents of children with type 1 diabetes |
| Buckloh 2008 | Diabetes complications in youth: Qualitative analysis of parents' perspectives of family learning and knowledge |
| Burckhardt 2019 | Use of remote monitoring with continuous glucose monitoring in young children with type 1 diabetes: The parents' perspective |
| Butler 2020 | Barriers and facilitators to involvement in children’s diabetes management among minority parents |
| Capistrant 2017 | Mental health and well-being among type 1 diabetes caregivers in India: Evidence from the IDREAM study |
| Castensøe‐Seidenfaden 2017 | Isolated thoughts and feelings and unsolved concerns: Adolescents’ and parents’ perspectives on living with type 1 diabetes—a qualitative study using visual storytelling |
| Chaney 1997 | Transactional patterns of child, mother, and father adjustment in insulin-dependent diabetes mellitus: A prospective study |
| Charron-Prochownik 2000 | Maternal health-related coping patterns and health and adjustment outcomes in children with type 1 diabetes |
| Cline 2011 | A pilot study of acute stress symptoms in parents and youth following diagnosis of type I diabetes |
| Costa-Cordella 2020 | Mentalizing in mothers and children with type 1 diabetes |
| Cox 2021 | Experience of caring for a child with type 1 diabetes mellitus in a food-insecure household: A qualitative evaluation |
| Crocket 2021 | Parental experiences of short term supported use of a do-it-yourself continuous glucose monitor (DIYRTCGM): A qualitative study |
| Cunningham 2011 | From caregiver psychological distress to adolescent glycemic control: The mediating role of perceived burden around diabetes management |
| Dehn-Hindenberg 2021 | Long-term occupational consequences for families of children with type 1 diabetes: The mothers take the burden |
| Elbalshy 2020 | Exploring parental experiences of using a do-it-yourself solution for continuous glucose monitoring among children and adolescents with type 1 diabetes: A qualitative study |
| Eshtehardi 2020 | On the money: Parental perspectives about finances and type 1 diabetes in youth |
| Feeley 2019 | Sleep in caregivers of children with type 1 diabetes |
| Feeley 2021 | Sleep in parental caregivers and children with type 1 diabetes |
| Fremont 2021 | Perspectives on remote glucose monitoring in youth with type 1 diabetes |
| Gallegos 2022 | A descriptive study of the quality of life and burden of mothers of children and adolescents with type 1 diabetes |
| Goldberg 2017 | Sense of coherence and parenting representation among parents of adolescents with type 1 diabetes |
| Grey 2011 | Coping skills training for parents of children with type 1 diabetes: 12-month outcomes |
| Grover 2016 | Coping and caregiving experience of parents of children and adolescents with type-1 diabetes: An exploratory study |
| Hamlett 1992 | Childhood chronic illness as a family stressor |
| Hanna 2000 | Parents' perceived benefits and barriers of adolescents' diabetes self-management: Part 2 |
| Hannonen 2015 | Psychological distress of children with early‐onset type 1 diabetes and their mothers' well‐being |
| Hanson 1988 | Father-absent adolescents with insulin-dependent diabetes mellitus: A population at risk? |
| Hansson 1994 | Parent-rated family climate: A concomitant to metabolic control in juvenile IDDM? |
| Harrington 2017 | Management and family burdens endorsed by parents of youth <7 years old with type 1 diabetes |
| Harris 1999 | Adolescents with diabetes from single-parent, blended, and intact families: Health-related and family functioning |
| Harrison 2020 | Parents’ relationship maintenance as a buffer for the stress of their adolescent’s type 1 diabetes |
| Haugstvedt 2011 | Perceived family burden and emotional distress: Similarities and differences between mothers and fathers of children with type 1 diabetes in a population-based study |
| Haugvik 2017 | 'My heart burns' – a qualitative study of perceptions and experiences of type 1 diabetes among children and youths in Tajikistan |
| Hawkes 2014 | Fear of hypoglycemia in parents of children with type 1 diabetes |
| Henderson 2021 | Diabetes-by-proxy: Virtual embodiment of disease by Oklahoma Choctaw parents of children with type 1 diabetes |
| Hoff 2005 | An intervention to decrease uncertainty and distress among parents of children newly diagnosed with diabetes: A pilot study |
| Holden 1997 | Controlling for general and disease-specific effects in child and family adjustment to chronic childhood illness |
| Hood 2007 | Updated and revised diabetes family conflict scale |
| Horsch 2012 | Cognitive and non-cognitive factors associated with posttraumatic stress symptoms in mothers of children with type 1 diabetes |
| Horsch 2007 | Anxiety, depressive, and posttraumatic stress symptoms in mothers of children with type 1 diabetes |
| Ingerski 2010 | Correlates of glycemic control and quality of life outcomes in adolescents with type 1 diabetes |
| Ivey 2009 | Finding the balance: Adolescents with type 1 diabetes and their parents |
| Jaser 2010 | A pilot study of observed parenting and adjustment in adolescents with type 1 diabetes and their mothers |
| Jaser 2014 | Coping and psychological distress in mothers of adolescents with type 1 diabetes |
| Jaser 2018 | Developing and testing an intervention to reduce distress in mothers of adolescents with type 1 diabetes |
| Joiner 2020 | Perceptions and experiences of living with type 1 diabetes among Latino adolescents and parents with limited English proficiency |
| Jonsson 2012 | The logic of care - parents' perceptions of the educational process when a child is newly diagnosed with type 1 diabetes |
| Jönsson 2016 | Parents HRQOL, their satisfaction with care, and children over the age of eight’s experiences of family support two years subsequent to the child’s diagnosis with type 1 diabetes |
| Kajale 2021 | Diabetes distress in Indian children with type 1 diabetes mellitus and their mothers |
| Keklik 2020 | Care burden and quality of life in mothers of children with type 1 diabetes mellitus |
| Khandan 2018 | Lived experiences of mothers with diabetic children from the transfer of caring role |
| Kobos 2017 | Diabetes, childcare, and performance of family functions |
| Kovacs 1990 | Psychological functioning among mothers of children with insulin-dependent diabetes mellitus: A longitudinal study |
| Landolt 2002 | Posttraumatic stress disorder in parents of children with newly diagnosed type 1 diabetes |
| Landolt 2005 | Prospective study of posttraumatic stress disorder in parents of children with newly diagnosed type 1 diabetes |
| Landolt 2003 | Incidence and associations of parental and child posttraumatic stress symptoms in pediatric patients |
| Landolt 2012 | The mutual prospective influence of child and parental post‐traumatic stress symptoms in pediatric patients |
| Law 2013 | Adolescent and parent diabetes distress in type 1 diabetes: The role of self-efficacy, perceived consequences, family responsibility and adolescent–parent discrepancies |
| Lewin 2005 | Validation of the pediatric inventory for parents in mothers of children with type 1 diabetes: An examination of parenting stress, anxiety, and childhood psychopathology |
| Lin 2008 | Mothers' experience supporting life adjustment in children with t1dm |
| Lindstrom 2017 | Mission impossible; the mothering of a child with type 1 diabetes - from the perspective of mothers experiencing burnout |
| Lindström 2016 | Group intervention for burnout in parents of chronically ill children—a small-scale study |
| Lopez 2008 | The relation between parental psychological distress and adolescent anxiety in youths with chronic illnesses: The mediating effect of perceived child vulnerability |
| Lowes 2005 | Newly diagnosed childhood diabetes: A psychosocial transition for parents? |
| Lowes 2004 | Childhood diabetes: Parents' experience of home management and the first year following diagnosis |
| Luo 2021 | Resilience outstrips the negative effect of caregiver burden on quality of life among parents of children with type 1 diabetes: An application of Johnson–Neyman analysis |
| Maas-van Schaaijk 2013 | The interrelationships among paternal and maternal parenting stress, metabolic control, and depressive symptoms in adolescents with type 1 diabetes mellitus |
| Mackey 2014 | Maternal depressive symptoms and disease care status in youth with type 1 diabetes |
| Majidi 2021 | Psychosocial outcomes in young adolescents with type 1 diabetes participating in shared medical appointments |
| Marker 2021 | Parent perspectives on educational and psychosocial intervention for recent-onset type 1 diabetes in their school-age child: A qualitative study |
| Marshall 2009 | Living with type 1 diabetes: Perceptions of children and their parents |
| Mello 2017 | The stress and coping context of type 1 diabetes management among Latino and non-Latino white early adolescents and their mothers |
| Moreira 2013 | Caring for a child with type 1 diabetes: Links between family cohesion, perceived impact, and parental adjustment |
| Moreira 2014 | Family cohesion and health-related quality of life of children with type 1 diabetes: The mediating role of parental adjustment |
| Morone 2021 | Influence of social determinants of health barriers to family management of type 1 diabetes in black single parent families: A mixed methods study |
| Morrow 2021 | Sleep habits of early school-aged children with type 1 diabetes and their parents: Family characteristics and diabetes management |
| Moyer 1989 | Caring for a child with diabetes: The effect of specialist nurse care on parents' needs and concerns |
| Mueller-Godeffroy 2018 | Psychosocial benefits of insulin pump therapy in children with diabetes type 1 and their families: The pumpkin multicenter randomized controlled trial |
| Muller-Godeffroy 2009 | Investigation of quality of life and family burden issues during insulin pump therapy in children with type 1 diabetes mellitus - Tempa large-scale multicenter pilot study |
| Ness 2020 | Challenges experienced by parents of emerging young adults with type 1 diabetes mellitus during the transition to college |
| Ng 2019 | Continuous glucose monitoring in children with type 1 diabetes improves well-being, alleviates worry and fear of hypoglycemia |
| Nguyen 2021 | Depression and anxiety in adolescents with type 1 diabetes and their parents |
| Nicholas 2013 | Examining preferences for website support to parents of adolescents with diabetes |
| Northam 1996 | Psychosocial and family functioning in children with insulin-dependent diabetes at diagnosis and one year later |
| Noser 2019 | Parental depression and diabetes-specific distress after the onset of type 1 diabetes in children |
| Noueiri 2018 | Impact of diabetes mellitus type 1 on Lebanese families' quality of life |
| Oron 2014 | A remote monitoring system for artificial pancreas support is safe, reliable, and user friendly |
| Oskouie 2013 | Mediating factors of coping process in parents of children with type 1 diabetes |
| Overgaard 2020 | Disruption, worries and autonomy in the everyday lives of adolescents with type 1 diabetes and their family members: A qualitative study of intrafamilial challenges |
| Palmer 2021 | Diabetes device downloading: Benefits and barriers among youth with type 1 diabetes |
| Palmer 2021 | Understanding the lived experience of children with type 1 diabetes in Kenya: Daily routines and adaptation over time |
| Palmer 2004 | The role of autonomy and pubertal status in understanding age differences in maternal involvement in diabetes responsibility across adolescence |
| Pate 2019 | Fear of hypoglycemia, anxiety, and subjective well-being in parents of children and adolescents with type 1 diabetes |
| Perez 2019 | Communicatively exploring uncertainty management of parents of children with type 1 diabetes |
| Polonsky 2020 | Impact of real-time CGM data sharing on quality of life in the caregivers of adults and children with type 1 diabetes |
| Pritlove 2020 | Experiences and perspectives of the parents of emerging adults living with type 1 diabetes |
| Rankin 2015 | Parents' experiences of managing their child's diabetes using an insulin pump: A qualitative study |
| Rankin 2016 | Parents' information and support needs when their child is diagnosed with type 1 diabetes: A qualitative study |
| Ridge 2014 | Diabetes-oriented learning family intervention (DOLFIN): A feasibility study evaluating an intervention for carers of young persons with type 1 diabetes |
| Rifshana 2017 | The parental experience of caring for a child with type 1 diabetes |
| Rodrigue 1994 | Parenting satisfaction and efficacy among caregivers of children with diabetes |
| Rohan 2014 | Identification and prediction of group-based glycemic control trajectories during the transition to adolescence |
| Saghaei 2017 | The effectiveness of cognitive-function stress management training in glycemic control in children and in mental health of mother caring for child with type 1 diabetes mellitus |
| Sassmann 2012 | Reducing stress and supporting positive relations in families of young children with type 1 diabetes: A randomized controlled study for evaluating the effects of the DELFIN parenting program |
| Schmidt 2012 | In-hospital survival skills training for type 1 diabetes: Perceptions of children and parents |
| Seiffge-Krenke 2002 | 'Come on, say something, dad!': Communication and coping in fathers of diabetic adolescents |
| Shah 2019 | Social network factors and anxiety among adolescents with type 1 diabetes and their parents |
| Smaldone 2011 | Perceptions of parenting children with type 1 diabetes diagnosed in early childhood |
| Souris 2021 | 'La Vida normal': Young people adapting to type 1 diabetes in Bolivia |
| Stanek 2020 | Stressful life events, parental psychosocial factors, and glycemic management in school-aged children during the 1 year follow-up of new-onset type 1 diabetes |
| Stewart 2000 | Pathways from emotional adjustment to glycemic control in youths with diabetes in Hong Kong |
| Stoppelbein 2007 | Brief report: The risk of posttraumatic stress disorder in mothers of children diagnosed with pediatric cancer and type 1 diabetes |
| Streisand 2005 | Pediatric parenting stress among parents of children with type 1 diabetes: The role of self-efficacy, responsibility, and fear |
| Sullivan-Bolyai 2014 | Engaging teens and parents in collaborative practice: Perspectives on diabetes self-management |
| Sullivan-Bolyai 2015 | Pets-d: Impact on diabetes management outcomes |
| Turin 2021 | Carer's attachment anxiety, stressful life-events and the risk of childhood-onset type 1 diabetes |
| Vervoort 2011 | Child's and parents' catastrophizing about pain is associated with procedural fear in children: A study in children with diabetes and their mothers |
| Vesco 2018 | Parent–adolescent dyadic diabetes distress: Associations with a1c and diabetes-related strengths |
| Vesco 2018 | Continuous glucose monitoring associated with less diabetes-specific emotional distress and lower a1c among adolescents with type 1 diabetes |
| Walker 1992 | Family resources and stress: A comparison of families of children with cystic fibrosis, diabetes, and mental retardation |
| Whittemore 2018 | Development of an e-health program for parents of adolescents with type 1 diabetes |
| Wiebe 2005 | Children's appraisals of maternal involvement in coping with diabetes: Enhancing our understanding of adherence, metabolic control, and quality of life across adolescence |
| Wu 2014 | Autonomy support and responsibility-sharing predict blood glucose monitoring frequency among youth with diabetes |
| Wysocki 2008 | Randomized, controlled trial of behavioral family systems therapy for diabetes: Maintenance and generalization of effects on parent-adolescent communication |
| Yi-Frazier 2018 | Trajectories of acute diabetes-specific stress in adolescents with type 1 diabetes and their caregivers within the first year of diagnosis |
| Yi-Frazier 2021 | The clock is ticking: Parental stress around emerging adulthood for adolescents with type 1 diabetes |
| Youngkin 2020 | Continuous glucose monitoring decreases hypoglycemia avoidance behaviors, but not worry in parents of youth with new onset type 1 diabetes |
