# Supplementary material for: Humanistic burden of pediatric type 1 diabetes on children and informal caregivers: systematic literature reviews
Source: Diabetol Metab Syndr. 2024 Mar 21;16:73. doi: 10.1186/s13098-024-01310-2 (PMC10956250; doi:10.1186/s13098-024-01310-2)
Supplement: Supplementary file 4 — Additional File 4: Quality assessment (Summary of quality assessment of included studies from the systematic literature reviews) [file 13098_2024_1310_MOESM4_ESM.docx]

# Additional File 4: Quality assessment

**Quality Assessment of Studies Reporting on the Burden of T1D in Children**

- Sixty-five studies were assessed with the Joanna Briggs Institute Quality Assessment Tool. Most studies clearly described inclusion criteria, measured outcomes in a valid and reliable way, and used an appropriate method of analysis. Study subjects and setting were described in detail for about half of the studies. The risk of bias was unclear for the measurement of exposure, measurement of the condition, and confounding factors.
- Eleven studies were assessed with the Newcastle-Ottawa Scale. Overall, studies scored 2 to 4 points out of a maximum of 9 points. For the selection domain, most studies scored 0-2 points (maximum 4 points). Most studies scored 0 (n = 5) and 2 (n = 4) out of 2 points for the comparability domain. Most studies scored 1 out of 3 points for the outcome domain (n = 8).

**Quality Assessment of Studies Reporting on the Burden of T1D in Informal Caregivers**

- Forty-two studies were assessed with the Joanna Briggs Institute Quality Assessment Tool. Most studies clearly described study setting and inclusion criteria and used an appropriate method of analysis. It was unclear if objective, standard criteria were used to measure the condition of “being a caregiver”.
- Seven studies were assessed with the Newcastle-Ottawa Scale. Overall, studies scored 2 to 6 points out of a maximum of 9 points. Most studies scored 1 (n = 4) or 2 (n = 2) point(s) for the selection domain (maximum 4 points). Three studies scored 0, while 4 studies scored 2 out of 2 points for the comparability domain. Most studies scored 1 out of 3 points for the outcome domain (n = 5).
- Four studies were assessed with Cochrane Risk of Bias version 2. Most studies showed some concerns of bias (n = 3) overall. All studies showed low risk of bias for missing outcome data, while all studies showed some concerns of bias for the measurement of the outcome. Most studies showed some concerns for deviations from the intended interventions (n = 3) and selection of the reported result (n = 2).
